# Thermal Solution Depolymerization of RAFT Telechelic Polymers

**DOI:** 10.1021/acsmacrolett.4c00286

**Published:** 2024-06-10

**Authors:** Nethmi De Alwis Watuthanthrige, Richard Whitfield, Simon Harrisson, Nghia P. Truong, Athina Anastasaki

**Affiliations:** † Laboratory of Polymeric Materials, Department of Materials, ETH Zurich, Zurich, 8093, Switzerland; ‡ Laboratoire de Chimie des Polymères Organiques, University of Bordeaux/Bordeaux-INP/CNRS UMR5629, Pessac 33607, France

## Abstract

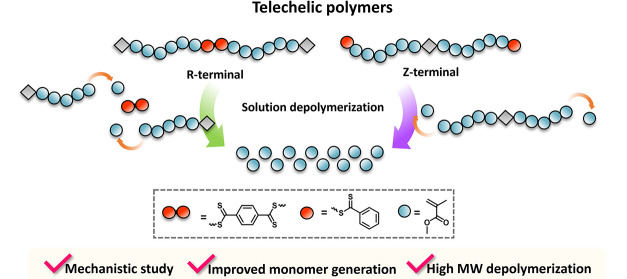

Thermal solution depolymerization is a promising low-temperature chemical recycling strategy enabling high monomer recovery from polymers made by controlled radical polymerization. However, current methodologies predominantly focus on the depolymerization of monofunctional polymers, limiting the material scope and depolymerization pathways. Herein, we report the depolymerization of telechelic polymers synthesized by RAFT polymerization. Notably, we observed a significant decrease in the molecular weight (*M*_n_) of the polymers during monomer recovery, which contrasts the minimal *M*_n_ shift observed during the depolymerization of monofunctional polymers. Introducing Z groups at the center or both ends of the polymer resulted in distinct kinetic profiles, indicating partial depolymerization of the bifunctional polymers, as supported by mathematical modeling. Remarkably, telechelic polymers featuring R-terminal groups showed up to 68% improvement in overall depolymerization conversion compared to their monofunctional analogues, highlighting the potential of these materials in chemical recycling and the circular economy.

The labile end-groups installed by reversible deactivation radical polymerization (RDRP), also referred to as controlled radical polymerization, enable the efficient depolymerization of polymers at temperatures significantly lower than those employed in pyrolysis.^1−4^ Over the past few years, several groups have contributed to the chemical recycling of polymers made by RDRP, typically by leveraging thermodynamically favorable conditions (i.e., heat and dilution).^3,5−8^ Research in this area was initiated by the groups of Gramlich and Raus, who highlighted the importance of selecting suitable reaction conditions to avoid competing depolymerization equilibria during the polymerization of bulky monomers.^9,10^ Matyjaszewski and co-workers also reported on the chemical recycling of bulky polymers, achieving high monomer regeneration.^11^ Ouchi and co-workers then illustrated the first depolymerization of a non-bulky polymer (i.e., poly(methyl methacrylate) by employing ruthenium catalysis, although relatively low conversions were achieved (e.g., 20%) and the depolymerizations were accompanied by side reactions.^12^ The group of Matyjaszewski was then able to suppress the side reactions, namely lactonization, and demonstrated the possibility of depolymerizing chlorine-containing polymers synthesized by atom transfer radical polymerization (ATRP) at 170 °C in the presence of either copper or iron.^13,14^ Our group subsequently demonstrated the depolymerization of bromine-terminated ATRP polymers by favoring activation over termination and lactonization also at 170 °C.^15^

On the other hand, reversible addition–fragmentation chain transfer (RAFT)-synthesized polymers require slightly lower temperatures (i.e., 120 °C) to trigger an efficient chain-end depolymerization in solution. For example, our group showed that a range of polymethacrylates, as well as insoluble polymer networks, could be rigorously depolymerized at 120 °C (with a 5 mM repeat unit concentration) reaching near-quantitative depolymerization while a unimer RAFT agent could also be retrieved.^16−19^ These temperatures can be further lowered when heat is combined with light, and a number of photothermal depolymerizations have recently been reported by the Summerlin group and our group independently, yielding faster depolymerization rates and overall higher depolymerization conversions.^20−23^

Bulk depolymerizations require much higher operating temperatures (∼220 °C), as the dilution effect can no longer be applied.^24,25^ Here, the incorporation of specific chain-ends, pendant groups, or the addition of a catalyst have been shown to enhance the depolymerization yields for both ATRP and RAFT-synthesized materials.^24−28^ However, the overwhelming majority of current depolymerization methodologies concern monofunctional polymers, with the only exception being a recent bulk depolymerization approach reported by the group of Sumerlin, whereby utilizing a difunctional photoiniferter, i.e., a combination of a trithiocarbonate ω-end and a phthalimide α-end, high degrees of depolymerization could be achieved.^24^ Inspired by this pioneering work, and recognizing the broad applications of telechelic polymers in self-assembly,^29,30^ material property fine-tuning,^31,32^ and bioconjugation,^33^ we report here the first thermal solution depolymerization of RAFT-synthesized linear telechelic polymers by employing either the Z or the R approach, as depicted in Figure 1. We particularly focused on the depolymerization kinetics of linear telechelic polymers in solution and developed a mathematical model to shed new light on their depolymerization pathways.

**Figure 1:**
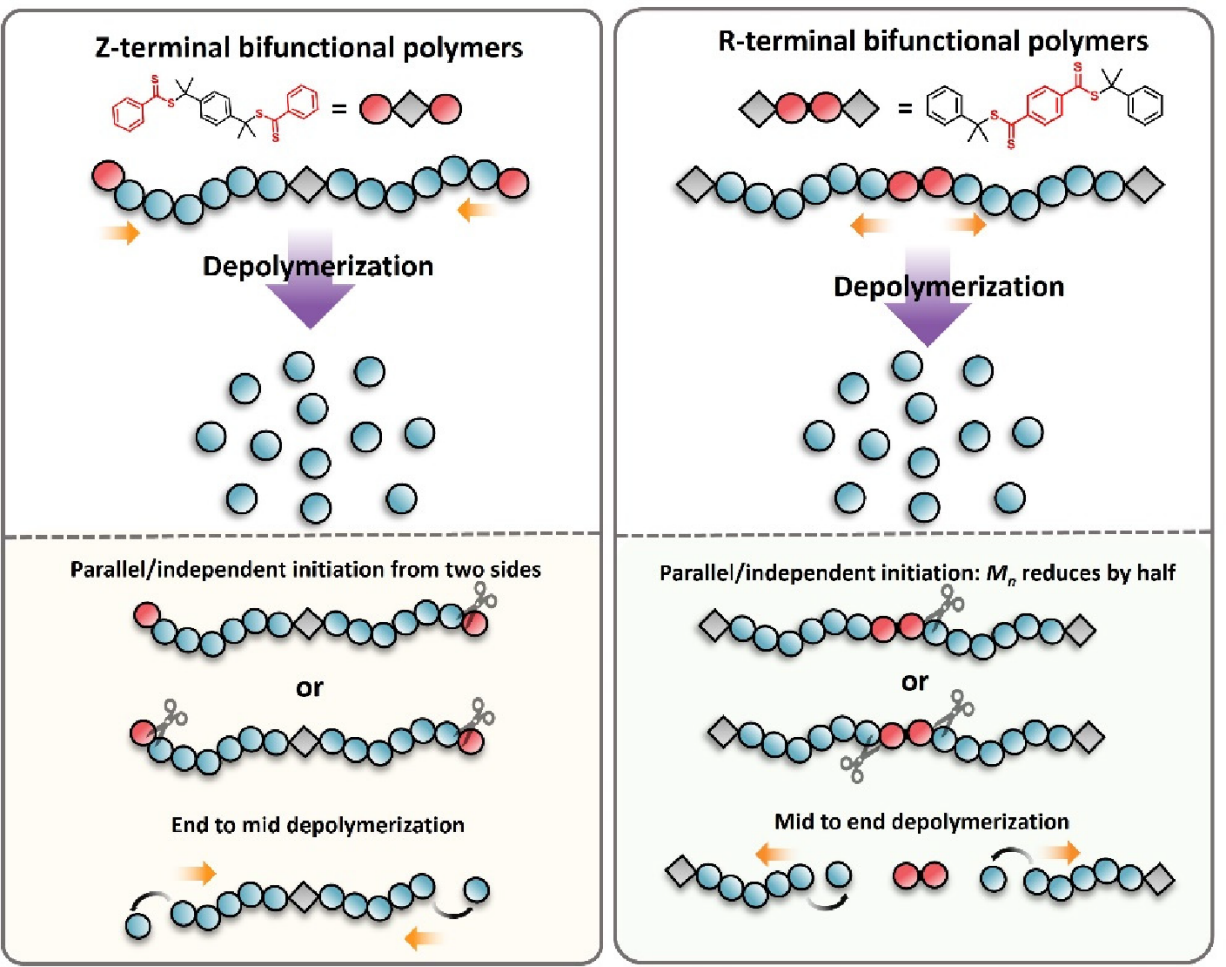
Conceptual scheme displaying the depolymerization of two distinct types of bifunctional polymers: Z-terminal bifunctional (left) and R-terminal bifunctional (right).

To synthesize the polymers, we followed both Z and R approaches, akin to the core-first approaches used in star polymer synthesis,^34−39^ utilizing two distinct types of bifunctional chain transfer agents (CTAs, Figures S1–S6). The Z-terminal bifunctional CTA, namely, 1,4-phenylenebis(propane-2,2-diyl) dibenzodithiate, possesses two Z groups symmetrically located at each chain-end.^40−43^ Upon polymerization, the distance between the two Z groups is determined by the targeted degree of polymerization (DP). Meanwhile, R-terminal bifunctional polymers contain two central Z groups and were synthesized using bis(2-phenylpropan-2-yl) benzene-1,4-bis(carbodithioate) as the CTA (Figure 1). Compared to their monofunctional analogues, both the Z and R terminal bifunctional polymers contain twice the amount of RAFT end-groups, thus potentially providing a significant advantage in the initiation of the depolymerization through activation of labile groups.^44^ In addition, Z-terminal bifunctional polymers are expected to either simultaneously depolymerize from both chain-ends or partially depolymerize from one chain-end, followed by depolymerization from the other chain-end at a later stage. In both cases, an end-to-middle depolymerization strategy is envisioned. Similarly, R-terminal bifunctional polymers are anticipated to either depolymerize at the same time from both the Z groups at the chain ends or to first partially depolymerize from one Z group followed by the depolymerization from the other Z group at a later stage. A significant difference here is that the initial Z group cleavage will result in the instantaneous decrease of the *M*_n_ triggering a mid-to-end depolymerization (Figure 1).

To initiate our study, a wide range of Z-terminal bifunctional polymers were synthesized (Table S1) via an optimized RAFT polymerization protocol, obtaining various DPs (i.e., DP = 75, 125, 240, 350, and 550).^40^ In all cases, detailed kinetic studies were conducted under optimal conditions (5 mM repeat unit concentration, 120 °C), inspired by a previous publication of our group concerning the depolymerization of monofunctional polymers.^16^ High depolymerization yields (up to 89%) were observed, as confirmed by both nuclear magnetic resonance (NMR) and size exclusion chromatography (SEC, Figure 2a,b). However, in contrast to the depolymerization of monofunctional polymers, a noticeable decrease in molecular weight was observed throughout the depolymerization of Z-terminal bifunctional polymers (Figures 2c and S7). For instance, with a starting molecular weight of 24000 (DP = 240), at 16% of depolymerization conversion, a molecular weight of 20000 was obtained (*M*_n_ shift = 17%; defined as the percentage decrease in *M*_n_ compared to the original *M*_n_ (*M*_n(0)_) of the polymer: *M*_n_ shift = (*M*_n(0)_ – *M*_n(*t*)_)/*M*_n(0)_ × 100%)). Interestingly, this trend continued also at higher conversions with the *M*_n_ decreasing to 18400 (*M*_n_ shift = 24%) when 24% of monomer recovery was achieved. Instead, and in line with previous observations,^18^ depolymerization of the analogous monofunctional polymers led to minimal molecular weight shift in comparable conversions due to rapid unzipping of the polymer chains upon chain-end activation (Table S2 and Figure S8). Such decrease in *M*_n_ could be correlated to enhanced deactivation during the depolymerization of telechelic polymers due to the higher CTA content.^17^ However, this possibility was excluded by control experiments whereby further addition of external CTA did not result in a meaningful enhancement of the *M*_n_ shift or the final extent of depolymerization (Figures S9 and S10).^17^ In addition, at higher depolymerization conversions, the decrease in *M*_n_ gradually became less pronounced. For example, at 45%, 63%, and 80% of monomer recovery, the *M*_n_ shifts were 30%, 40%, and 46%, respectively, thus further indicating that a controlled depolymerization had not occurred.

**Figure 2:**
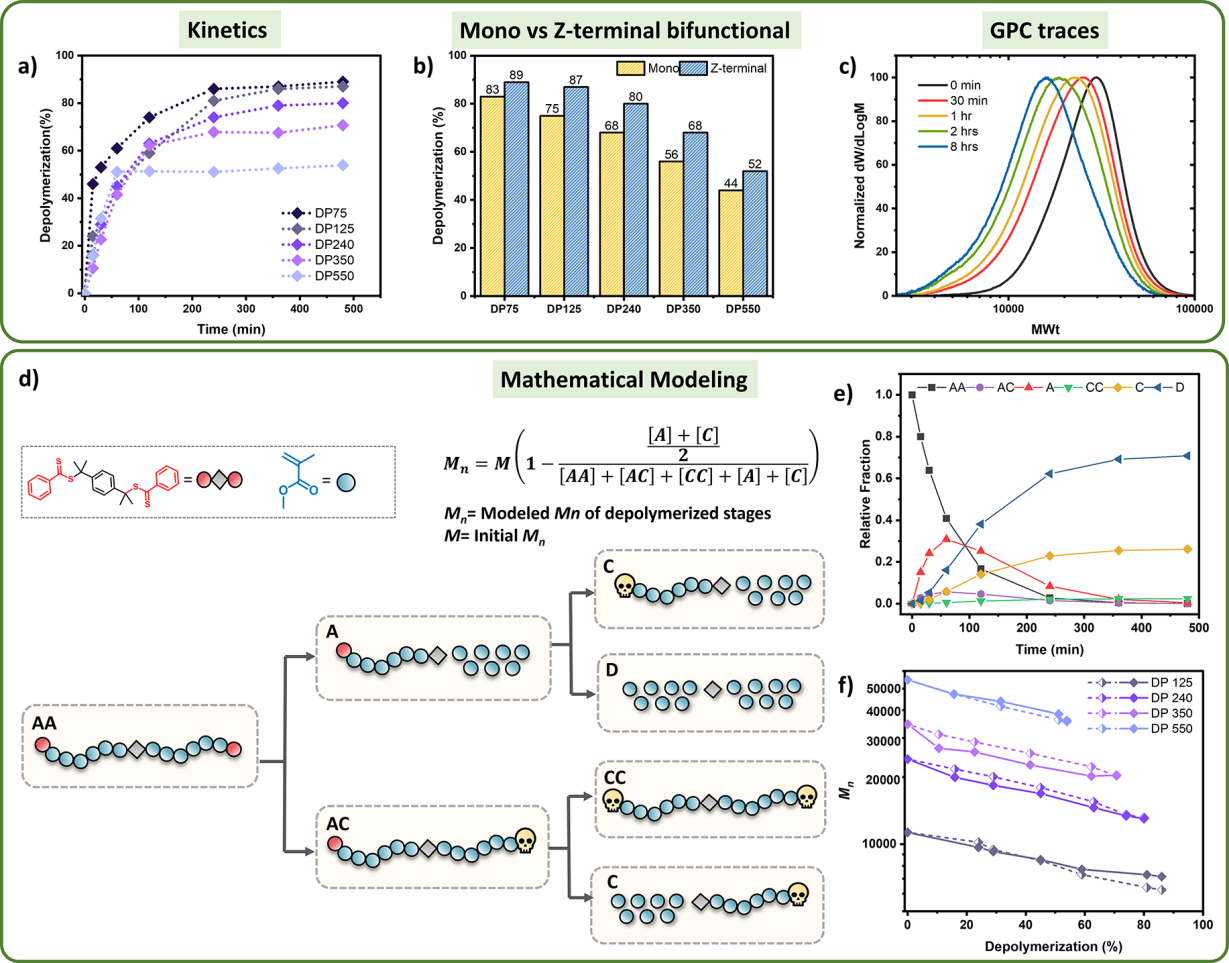
(a) Depolymerization kinetics for DP 75, 125, 240, 350, and 550 (5 mM repeat unit concentration, 120 °C in dioxane); (b) Comparison of the final depolymerization conversions of monofunctional and Z-terminal bifunctional polymers; (c) SEC traces of the DP 240 Z-terminal bifunctional polymer upon depolymerization at 0, 30, 60, 120, and 480 min; (d) Schematic diagram describing the pathways during the depolymerization of the Z-bifunctional polymers: AA: Polymer with two living chain ends (*M*_n_ = *M*), AC: polymer with one living and one dead chain end, (*M*_n_ = *M*), CC: polymer with 2 dead chain ends, (*M*_n_ = *M*), A: polymer with one living chain end (after depolymerization of one branch), (*M*_n_ = *M*/2), C: polymer with one dead chain end (after depolymerization of one branch) (*M*_n_ = *M*/2); (e) Relative fractions of each species resulting from the depolymerization of Z-terminal bifunctional polymers with DP 240; (f) Fit between the modeled and experimental *M*_n_ values during the depolymerization process (dashed lines represent the *M*_n(model)_ and solid lines represent the *M*_n(exp)_).

To elucidate the observed reduction in molecular weight during the depolymerization, we employed mathematical modeling based on the data obtained from SEC analysis (see Supporting Information, Figure S11). Our modeling took into account the prevalence of each species potentially produced during the depolymerization and their associated molecular weights. The initial polymer chain features living Z groups at both chain-ends (AA, Figure 2d), and upon the first activation, either partial depolymerization (A) or instantaneous termination will occur (AC). The partially depolymerized chain A can subsequently undergo termination (C) or a complete depolymerization (D) whereby all the monomer is released. Fully depolymerized chains (D) are excluded from our molecular weight distribution modeling as the initial chain has entirely converted into monomers. The AC species can, upon a subsequent activation event, either again terminate (CC), whereby the initial molecular weight is maintained, or partially depolymerize from the remaining active chain-end (C). Rate equations for each event were formulated based on SEC analysis, and equations for the concentrations of each species were derived. As shown in Figure 2e, the initial AA species gradually reduced to zero as the depolymerization proceeds. Instead, the concentration of AC species remains relatively constant during depolymerization, suggesting that negligible termination occurs at the very beginning of the reaction. This data are also in line with our UV data, showing an analogous consumption of the RAFT end-group during the depolymerization. The A species first increases in concentration, rationalized by the initial partial depolymerization, and then gradually disappears due to the formation of either C or D species. C species increase with time, reflecting potential termination events during the second activation event. Finally, CC species do not seem to be formed at an appreciable level, suggesting that termination of both chain-ends is unlikely. Figure 2f illustrates the evolution of *M*_n_ values for Z-terminal bifunctional polymers. A very good agreement is observed for the higher DPs (e.g., DP = 125, 240, 350, and 550). This alignment between modeled and experimental *M*_n_ values is also evident for other methacrylate polymers, such as poly(benzyl methacrylate) and poly(butyl methacrylate) (Figure S11). However, for shorter chains (e.g., DP = 75), a relatively lower extent of correlation is observed (Figure S13). This is presumably due to side reactions associated with the higher radical concentrations, for example, termination between two active chains or an increased extent of deactivation at these lower molecular weights.

Next, the depolymerization of the R-terminal bifunctional polymers was examined. Different DPs were also targeted (DP = 75, 125, 240, 350, 550, and 680), and the materials were rigorously purified prior to use (Table S1). To verify the symmetric nature of the obtained polymers, end-group removal experiments were performed,^45,46^ resulting in the efficient cleavage of the initial polymer chain into two segments (Figure S14). Detailed kinetic analysis revealed higher depolymerization yields with this approach, as opposed to the monofunctional analogues (Figure 3b). For example, for DP = 240 (*M*_n_ = 23700), 84% of depolymerization was achieved, while the monofunctional equivalent only led to 68% of depolymerization. In a similar fashion, for DP = 550 (*M*_n_ = 55000), the depolymerization of the telechelic polymers reached 68% as opposed to only 44% achieved with the monofunctional materials. It is noted that lower conversions were obtained in all cases for higher DPs, in line with previous depolymerization reports.^18^ Considering the success of this approach, we also attempted the depolymerization of an even higher molecular weight telechelic material (DP = 680, *M*_n_ ∼ 68000) and up to 57% of monomer was recovered. Instead, the monofunctional analogue of comparable molecular weight resulted in only 34% conversion (Figure 3c). These observations suggest that a substantial (up to 68%) improvement in depolymerization yield is possible when telechelic polymers are employed. It is also noted that previous reports focusing on the solution depolymerization of monofunctional polymers with higher molecular weights could not reach a high extent of monomer recovery, further highlighting the potential of this approach.^18^ Importantly, despite using identical experimental conditions (repeat unit concentration and temperature), R-terminal polymers exhibited superior depolymerization compared to both monofunctional and Z-terminal polymers. This improved depolymerization efficiency may be attributed to the presence of interconnected dithiobenzoate groups (through a phenyl ring) within the backbone. These dithiobenzoate groups can mutually influence each other’s activation, enhancing the overall depolymerization process.

**Figure 3:**
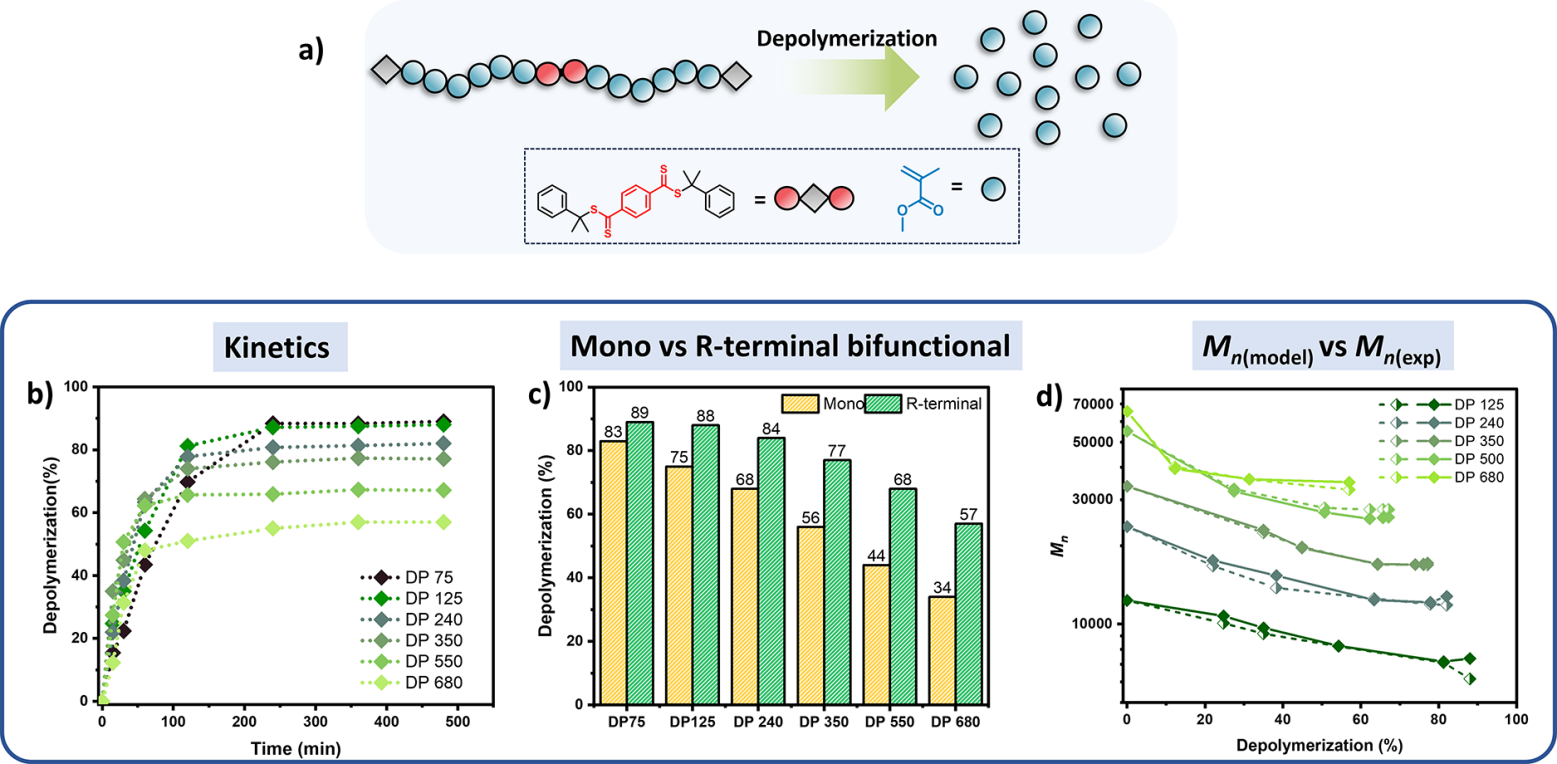
(a) Schematic representation of the depolymerization of R-terminal bifunctional PMMA polymers; (b) Depolymerization kinetics for DP 75, 125, 240, 350, 550, and 680 (5 mM, 120 °C in dioxane); (c) Final depolymerization conversions for mono and R-terminal bifunctional polymers with DP 75, 125, 240, 350, 550, and 680; (d) Fit between the modeled and experimental *M*_n_ values during the depolymerization process (dashed lines represents the *M*_n(model)_ and solid lines represent the *M*_n(exp)_).

Successively, detailed kinetics for the R-terminal polymers were conducted using a polymer of DP = 240 as the model telechelic material. Interestingly, upon depolymerization to 38% conversion, a higher molecular weight decrease was recorded by SEC (*M*_n_ shift = 35%) for the R-terminal polymers. This is in contrast to the Z-terminal materials, whereby at 45% conversion, a *M*_n_ shift of only 30% was achieved (Table S3). This trend was also continued at higher monomer conversions, and when 63% monomer was retrieved, the final *M*_n_ shift of 48% was already reached. Instead, for the Z-terminal polymers at comparable conversions (i.e., 63%) only 40% of *M*_n_ shift could be obtained (Figures S15 and S16). These findings suggest that for the R-terminal bifunctional polymers, a greater shift in *M*_n_ takes place as even a single Z group activation will result in chain scission and subsequent halving of the molecular weight, regardless of whether any depolymerization then occurs. Further depolymerization conversions (i.e., up to 84%) did not affect the *M*_n_. To provide a more quantitative explanation of the depolymerization pathways, a slightly modified mathematical model was then applied to the R-terminal polymers, as summarized in SI. Despite the complexity of the depolymerization mechanism in this system, Figure 3d shows a strong agreement between experimental and modeled values, in line with our observations by SEC analysis (Figure S17).

In conclusion, we investigated the thermal solution depolymerization of telechelic polymers synthesized by RAFT polymerization. In particular, the depolymerization of both Z- and R-terminal bifunctional materials was examined. In contrast to the depolymerization of monofunctional polymers, the depolymerization of telechelic polymers revealed a unique kinetic profile. As the monomer was regenerated, a significant molecular weight decrease was observed for both telechelic materials, and this decrease was more pronounced for the R-terminal analogues. This decrease was verified through a mathematical model and was attributed to the partial depolymerization of the bifunctional polymers. Notably, the R-terminal bifunctional polymers could be depolymerized to much higher conversions when compared with their monofunctional counterparts, paving the way for further exciting opportunities in chemical recycling of high molecular weight materials.

## Supporting Information Available

General information, experimental procedures, ^1^H NMR spectra, and SEC traces (PDF)
