# Supplementary material for: Thermal Solution Depolymerization of RAFT Telechelic Polymers
Source: ACS Macro Lett. 2024 Jun 10;13(7):806–11. doi: 10.1021/acsmacrolett.4c00286 (PMC11256755; doi:10.1021/acsmacrolett.4c00286)
Supplement: Supplementary file 1 — mz4c00286_si_001.pdf [file mz4c00286_si_001.pdf]

Supporting Information

Thermal Solution Depolymerization of RAFT Telechelic Polymers

Nethmi De Alwis Watuthanthrige[a], Richard Whitfield[a], Nghia P. Truong[a], Simon Harrisson[b], Athina Anastasaki[a,*]

[a] Laboratory of Polymeric Materials, Department of Materials, ETH Zurich, Zurich, 8093, Switzerland
[b] Laboratoire de Chimie des Polymères Organiques, University of Bordeaux/Bordeaux-INP/CNRS UMR5629, Pessac, France

Contents

1. Methods 3

1.1. Materials 3

1.2. NMR Spectroscopy 3

1.3. Size-exclusion chromatography (SEC) 3

2. Experimental 3

2.1. Synthesis of 1,4-Bis(2-(thiobenzoylthio)prop-2-yl)benzene (Z-terminal bifunctional CTA) 3

2.2. Synthesis of bis(2-phenylpropan-2-yl) benzene-1,4-bis(carbodithioate) (R-terminal bifunctional CTA) 4

2.3. Synthesis of PMMA polymers using Z-terminal bifunctional CTA 5

2.4. Synthesis of PMMA polymers using R-terminal bifunctional CTA 5

2.5. Synthesis of PMMA polymers using cumyl dithiobenzoate 6

2.6. Typical Depolymerization Procedure 6

2.7. Depolymerization with addition of CTA 7

2.8. End group removal experiment 7

3. Mathematical Modelling 7

3.1. Mathematical model for Z-terminal bifunctional polymers 8

3.2. Mathematical model for R-terminal bifunctional polymers 11

4. Supplementary Figures: 14

1. Methods

1.1. Materials

All materials were purchased from either Sigma Aldrich or Fischer Scientific unless otherwise stated.

1.2. NMR Spectroscopy

^{1}H-NMR spectra were recorded on a Bruker Avance-300 spectrometer using acetone-d6, dimethyl sulfoxide-d6, or $CDCl_3$ as the NMR solvent. Chemical shifts are given in ppm downfield from tetramethylsilane and referenced to residual solvent proton signals.

1.3. Size-exclusion chromatography (SEC)

SEC was measured on a Shimadzu equipment comprising a CBM-20A system controller, LC-20AD pump, SIL-20A automatic injector, 10.0 μm bead-size guard column (50 x 7.5 mm) followed by three KF-805L columns (300 x 8 mm, bead size: 10 μm, pore size maximum: 5000 Å), SPD-20A ultraviolet detector, and an RID-20A differential refractive index detector. The column temperature was maintained at 40 °C using a CTO-20A oven. The flow rate was set to 1 ml/min and with N, N-dimethylacetamide (DMAc, Acros, HPLC grade, with 0.03 w/v LiBr) as the eluent. Molecular weights were determined relative poly(methyl methacrylate) standards with molecular weights ranging from 5,000 to 1.5 x 10^6 g/mol (Agilent Technologies). All SEC samples were dissolved in DMAc and passed through 0.45 μm filters prior to analysis

2. Experimental

2.1. Synthesis of 1,4-Bis(2-(thiobenzoylthio)prop-2-yl)benzene (Z-terminal bifunctional CTA)

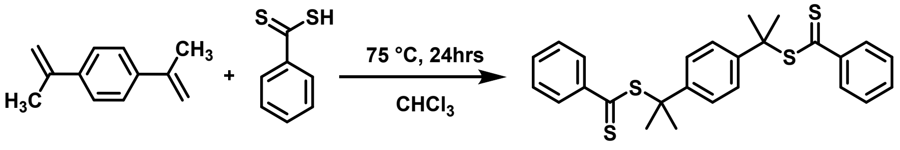

Scheme S 1: Synthesis of 1,4-Bis(2-(thiobenzoylthio)prop-2-yl)benzene

The synthesis of 1,4-Bis(2-(thiobenzoylthio)prop-2-yl)benzene was adapted from the literature with slight modifications.[1] A mixture of dithiobenzoic acid (6.12 g, 0.0397 mol) and 1,4-diisopropenylbenzene (3.00 g, 0.0189 mol) in chloroform (50 mL) was heated at 75 °C for 20 hours. The volatiles were removed under reduced pressure, and the residue was triturated with 1:2 diethyl ether/hexanes to isolate the product as a purple solid (41.1%). ^{1}H NMR, δ (ppm): 1.99 (12H, s); 7.33 (m, 4H); 7.47 (m, 2H); 7.50 (s, 4H) and 7.86 (m, 4H).

2.2. Synthesis of bis(2-phenylpropan-2-yl) benzene-1,4-bis(carbodithioate) (R-terminal bifunctional CTA)

The synthesis of bis(2-phenylpropan-2-yl) benzene-1,4-bis(carbodithioate) was inspired by the method outlined for 2-Phenylpropan-2-yl benzodithioate.[2]

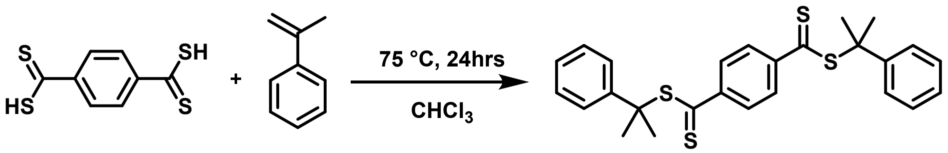

Scheme S 2: Synthesis of bis(2-phenylpropan-2-yl) benzene-1,4-bis(carbodithioate)

In 300 mL of dry methanol, a solution of sodium methoxide (10.8 g, 0.20 mol) was prepared and sulfur (6.40 g, 0.20 mol) was added. This was then refluxed for 3 hours, beforep-xylyl chloride (8.75 g, 0.05 mol) was added gradually over 1.5 hours. This mixture was then refluxed overnight. The resulting solution was filtered, and the solvent partially evaporated to yield a dark-brown residue. This residue was treated with a large excess of dilute HCl to precipitate the 1,4-Benzenedicarbodithioic acid (8.5 g).

Next, a mixture of 1,4-Benzenedicarbodithioic acid (3.00 g, 0.0130 mol) and α-methylstyrene (3.87 g, 0.0326 mol) in chloroform (50 mL) was heated at 75 °C for 20 hours. The reaction mixture was then extracted with sodium hydroxide solution to remove unreacted 1,4-Benzenedicarbodithioic acid. The remaining organic layer was concentrated using a rotary evaporator to remove the solvent. The resulting solid was washed with hexane and dissolved in a 1:1 mixture of ethyl acetate and hexane. This solution was then passed through a basic alumina column, and the volatiles were removed under reduced pressure to obtain a purple solid of bis(2-

phenylpropan-2-yl) benzene-1,4-bis(carbodithioate). The ^{1}H NMR spectrum (300 MHz, CDCl3) displayed peaks at δ (ppm): 2.02 (s, 12H), 7.34 (m, 4H), 7.49 (d, 6H), and 7.87 (m, 4H).

2.3. Synthesis of PMMA polymers using Z-terminal bifunctional CTA

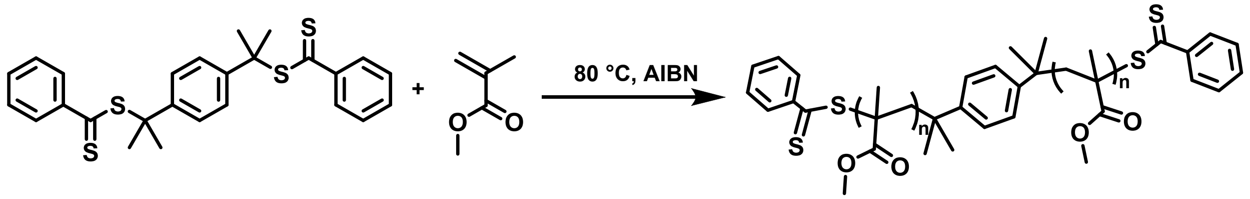

Scheme S 3 : Synthesis of R-terminal bifunctional polymers

In a 10 mL round bottom flask with a stirrer bar, 29.8 mg of 1,4-Bis(2-(thiobenzoylthio)prop-2-yl)benzene (1 equivalent, 0.064 mmol) dissolved in 500 µL of benzene was combined with 2.1 mg of AIBN (0.2 equivalents, 0.013 mmol) , and 2.00 g of methyl methacrylate (312.5 equivalents, 0.020 mol). The flask was sealed with a septum, and nitrogen bubbling for 15 minutes followed by polymerization in an oil bath at 80 °C. Samples were periodically withdrawn under a nitrogen blanket for ^{1}H-NMR analysis and filtered through a syringe filter (0.45 µM PTFE membrane) before SEC analysis. Polymerization was halted at 40% conversion by removing the reaction from the oil bath and removing the septum. The quantities specified here are for the synthesis of DP125 polymer, adjusted according to the targeted DPs.

2.4. Synthesis of PMMA polymers using R-terminal bifunctional CTA

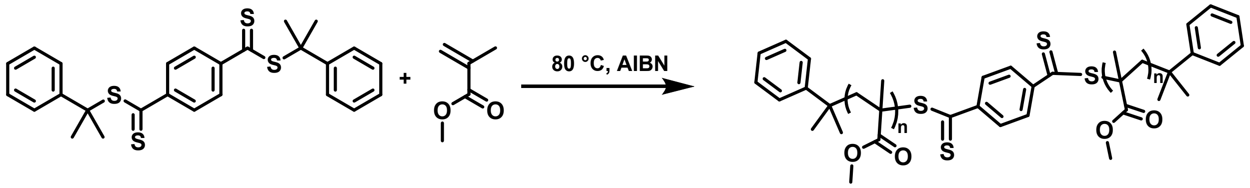

Scheme S 4: Synthesis of R-terminal bifunctional polymers.

In a 10 mL round bottom flask equipped with a stirrer bar, 29.8 mg bis(2-phenylpropan-2-yl) benzene-1,4-bis(carbodithioate) (1 equivalent, 0.064 mmol) dissolved in 500 µL of benzene was combined with 2.1 mg of AIBN (0.2 equivalents, 0.013 mmol) and 2.00 g of methyl methacrylate (312.5 equivalents, 0.020 mol) and 29.8 mg bis(2-phenylpropan-2-yl) benzene-1,4-bis(carbodithioate) (1 equivalent, 0.064 mmol). The flask was sealed with a septum, and

deoxygenation was carried out by bubbling nitrogen for 15 minutes before conducting the polymerization in an oil bath at 80 °C. Samples were periodically withdrawn under a nitrogen blanket for ^{1}H-NMR analysis and filtered through a syringe filter (0.45 μM PTFE membrane) before SEC analysis. Polymerization was halted at 40% conversion by removing the reaction from the oil bath and removing the septum. The quantities specified here are for the synthesis of DP125 polymer and were adjusted according to the targeted DPs.

2.5. Synthesis of PMMA polymers using cumyl dithiobenzoate

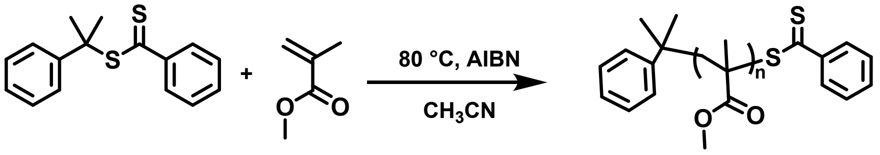

Scheme S 5: Synthesis of monofunctional polymers

In a 10 mL round bottom flask equipped with a stirrer bar, 2.1 mg of AIBN (0.2 equivalents, 0.013 mmol) was combined with 2.00 g of methyl methacrylate (312.5 equivalents, 0.020 mol), 17.4 mg of 2-Phenylpropan-2-yl benzodithioate (1 equivalent, 0.064 mmol) and 1.00 mL of Acetonitrile. The flask was then sealed with a septum, and deoxygenation was carried out by bubbling nitrogen for 15 minutes before conducting the polymerization in an oil bath at 80 °C. Samples were periodically withdrawn under a nitrogen blanket for ^{1}H-NMR analysis and filtered through a syringe filter (0.45 μM PTFE membrane) prior to SEC analysis. Polymerization was stopped at 40% conversion by removing the reaction from the oil bath and removing the septum. The quantities specified here are for the synthesis of DP125 polymer and were adjusted according to the targeted DPs.

2.6. Typical Depolymerization Procedure

In a 250 ml schlenk flask, 20 mg of PMMA was dissolved in 40 ml 1,4-dioxane (5 mM repeat unit concentration). 25μL of poly(ethylene glycol) monomethyl ether (M_n=350 Da) was then added as an internal standard for ^{1}H NMR analysis. The schlenk tube was sealed with a rubber septum and deoxygenated by nitrogen bubbling for 20 minutes. The schlenk flask was then placed in an oil bath at 120 °C (submerged ~2 cm below the surface of the oil bath) to start the reaction. To take samples, the reaction was periodically removed from the oil bath and quickly added to a water bath until the solution cooled to room temperature. The solution was then sampled under a nitrogen

blanket. For SEC samples, ~3 ml of the sample solution was blow-dried, dissolved in 1.5 ml DMAc, and passed through a syringe filter (0.45 µm PTFE membrane) prior to analysis.

2.7. Depolymerization with addition of CTA

In order to study the control of the depolymerization reactions, the DP 240 monofunctional polymer and Z-terminal polymer were mixed with 1 and 5 equivalents of chain transfer agent (cumyl dithiobenzoate) and carried out the depolymerization reactions using the same procedure stated in 2.6.

2.8. End group removal experiment

A 15 mL glass vial containing 50 mg of PMMA polymer was sealed with a rubber septum and deoxygenated for 15 minutes. The deoxygenated vial was then placed in an oil bath at 180°C and heated for 45 minutes. During this period, the pink-colored polymer turned into a yellow-colored solid, indicating the removal of the end group. The end group removal was subsequently characterized by SEC through the loss of the Z group UV signal.

3. Mathematical Modelling

Mathematical modeling was conducted utilizing data acquired through SEC analysis.

1. Depolymerization %= $\frac{RI\ Signal\ at\ time\ 0 - RI\ signal\ at\ time\ t}{RI\ Signal\ at\ time\ 0}$
2. UV loss %= $\frac{UV\ Signal\ at\ time\ 0 - UV\ signal\ at\ time\ t}{UV\ Signal\ at\ time\ 0}$

In formulating the mathematical model, we assumed that the chains undergo either instantaneous termination following the activation of the Z group or depolymerization until completion or until they encounter an end. The rate coefficient for depolymerization was designated as k_2, while the rate coefficient for termination was labeled as k_1. Based on these assumptions, rate equations were formulated for each species to track their concentrations throughout the process. The values of k_1 and k_2 were then precisely estimated to optimize the model's alignment with experimental data, particularly %depolymerization and %UV loss, minimizing discrepancies. Once the model achieved a superior fit, the optimized k_1 and k_2 values were applied to accurately determine the concentrations of each species at time t. Subsequently, the M_n was estimated utilizing the concentrations of each species.

3.1. Mathematical model for Z-terminal bifunctional polymers

Species:

AA : Polymer with 2 living chain ends, $M_n = M$

AC : Polymer with one living and one dead chain end, $M_n = M$

CC : Polymer with 2 dead chain ends, $M_n = M$

A : Polymer with one living chain end (after depolymerization of one branch), $M_n = M/2$

C : Polymer with one dead chain end (after depolymerization of one branch), $M_n = M/2$

Reactions:

AA --> AC (termination, rate coefficient k_1)

AA --> A (depolymerization, rate coefficient k_2)

AC --> CC (termination, rate coefficient k_1)

AC --> C (depolymerization, rate coefficient k_2)

A --> C (termination, rate coefficient k_1)

A --> D (depolymerization, rate coefficient k_2)

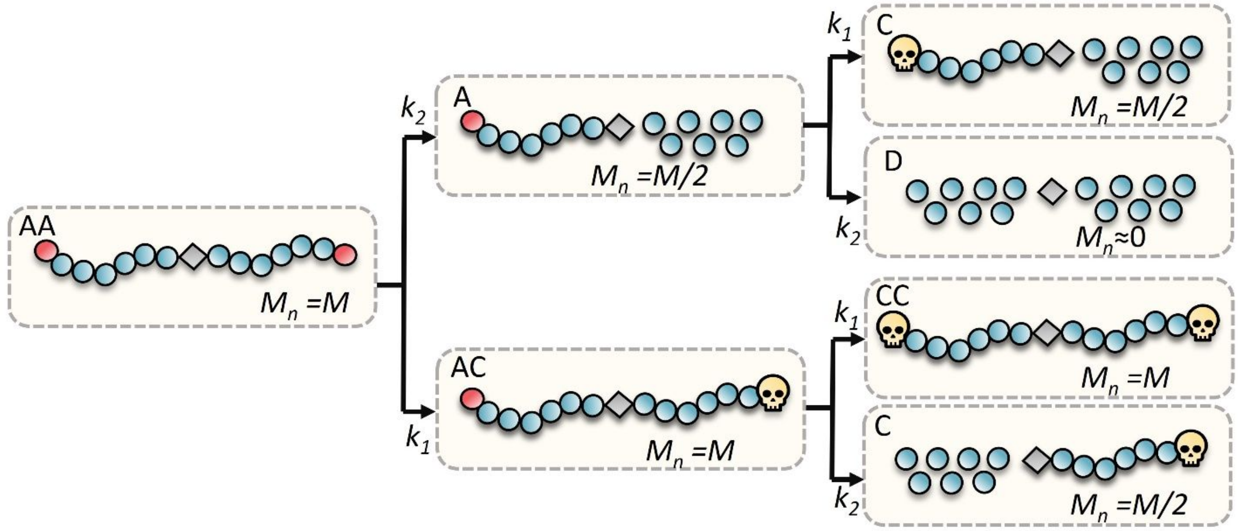

Scheme S6: Schematic diagram describing the events during the depolymerization of the Z-bifunctional polymers: AA : Polymer with 2 living chain ends (M_n =M) AC : Polymer with one living and one dead chain end, (M_n =M), CC : Polymer with 2 dead chain ends, (M_n =M), A : Polymer with one living chain end (after depolymerization of one

branch), (M_n =M /2), C : Polymer with one dead chain end (after depolymerization of one branch) (M_n =M /2), D : Depolymerization of both branches M_n = 0, k_1=rate of termination, k_2=rate of depolymerization

Kinetics:

$$\frac{-d[AA]}{dt} = k_1[AA] + k_2[AA]$$

$$\frac{d[AC]}{dt} = k_1[AA] - k_1[AC] - k_2[AC]$$

$$\frac{d[CC]}{dt} = k_1[AC]$$

$$\frac{d[A]}{dt} = k_2[AA] - k_1[A] - k_2[A]$$

$$\frac{d[C]}{dt} = k_1[A] + k_2[AC]$$

Solving the system of differential equations gives:

$$[AA] = [AA]_0 \mathrm{e}^{-(\mathrm{k_1}+\mathrm{k_2})t}$$

$$[AC] = k_1[AA]_0 t e^{-(k_1+k_2)t}$$

$$[CC] = \frac{k_1^2}{(k_1+k_2)^2}[AA]_0\left(1-(1+(k_1+k_2)t)e^{-(k_1+k_2)t}\right)$$

$$[A] = k_2[AA]_0 t e^{-(k_1+k_2)t}$$

$$[C] = \frac{2\,k_1k_2}{(k_1+k_2)^2}[AA]_0\left(1-(1+(k_1+k_2)t)e^{-(k_1+k_2)t}\right)$$

Average MW:

$$M_n = M\left(1-\frac{\frac{[A]+[C]}{2}}{[AA]+[AC]+[CC]+[A]+[C]}\right)$$

$$M_n = M\left(1-\frac{\frac{k_2}{2}te^{-(k_1+k_2)t}+\frac{k_1k_2}{(k_1+k_2)^2}\left(1-(1+(k_1+k_2)t)e^{-(k_1+k_2)t}\right)}{\mathrm{e}^{-(\mathrm{k_1}+\mathrm{k_2})t}+(k_1+k_2)te^{-(k_1+k_2)t}+\frac{\left(k_1^2+2\,k_1k_2\right)}{(k_1+k_2)^2}\left(1-(1+(k_1+k_2)t)e^{-(k_1+k_2)t}\right)}\right)$$

$$\frac{M_n}{M} = 1-\frac{\frac{k_2}{2}t+\frac{k_1k_2}{(k_1+k_2)^2}\left(e^{(k_1+k_2)t}-(1+(k_1+k_2)t)\right)}{1+(k_1+k_2)t+\frac{\left(k_1^2+2\,k_1k_2\right)}{(k_1+k_2)^2}\left(e^{(k_1+k_2)t}-(1+(k_1+k_2)t)\right)}$$

$$\frac{M_n}{M} = 1-\frac{\frac{k_2}{2}t+\frac{k_1k_2}{(k_1+k_2)^2}\left(e^{(k_1+k_2)t}-(1+(k_1+k_2)t)\right)}{\frac{k_2^2}{(k_1+k_2)^2}(1+(k_1+k_2)t)+\frac{\left(k_1^2+2\,k_1k_2\right)}{(k_1+k_2)^2}e^{(k_1+k_2)t}}$$

$$\frac{M_n}{M} = 1-\frac{(k_1+k_2)^2\frac{k_2}{2}t+k_1k_2\left(e^{(k_1+k_2)t}-(1+(k_1+k_2)t)\right)}{k_2^2(1+(k_1+k_2)t)+\left(k_1^2+2\,k_1k_2\right)e^{(k_1+k_2)t}}$$

$$\frac{M_n}{M} = \frac{k_2^2(1+(k_1+k_2)t)+\left(k_1^2+2\,k_1k_2\right)e^{(k_1+k_2)t}-(k_1+k_2)^2\frac{k_2}{2}t-k_1k_2\left(e^{(k_1+k_2)t}-(1+(k_1+k_2)t)\right)}{k_2^2(1+(k_1+k_2)t)+\left(k_1^2+2\,k_1k_2\right)e^{(k_1+k_2)t}}$$

$$\frac{M_n}{M} = \frac{(k_2^2 + k_1 k_2)(1 + (k_1 + k_2)t) + \left(k_1^2 + k_1 k_2\right) e^{(k_1+k_2)t} - (k_1 + k_2)^2 \frac{k_2}{2} t}{k_2^2 (1 + (k_1 + k_2)t) + \left(k_1^2 + 2\, k_1 k_2\right) e^{(k_1+k_2)t}}$$

$$\frac{M_n}{M} = \frac{\frac{k_2}{k_1 + k_2}(1 + (k_1 + k_2)t) + \frac{k_1}{k_1 + k_2} e^{(k_1+k_2)t} - \frac{k_2}{2} t}{\frac{k_2^2}{(k_1 + k_2)^2}(1 + (k_1 + k_2)t) + \left(1 - \frac{k_2^2}{(k_1 + k_2)^2}\right) e^{(k_1+k_2)t}}$$

$$\frac{M_n}{M} = \frac{\frac{k_2}{k_1 + k_2} + k_2 t + \frac{k_1}{k_1 + k_2} e^{(k_1+k_2)t} - \frac{k_2}{2} t}{\frac{k_2^2}{(k_1 + k_2)^2}(1 + (k_1 + k_2)t) + \left(1 - \frac{k_2^2}{(k_1 + k_2)^2}\right) e^{(k_1+k_2)t}}$$

$$\frac{M_n}{M} = \frac{\frac{k_2}{k_1 + k_2} + \frac{k_2}{2} t + \frac{k_1}{k_1 + k_2} e^{(k_1+k_2)t}}{\frac{k_2^2}{(k_1 + k_2)^2} + \frac{k_2^2 t}{k_1 + k_2} + \left(1 - \frac{k_2^2}{(k_1 + k_2)^2}\right) e^{(k_1+k_2)t}}$$

At t = 0: $\frac{M_n}{M} = \frac{\frac{k_2}{k_1 + k_2} + \frac{k_1}{k_1 + k_2}}{\frac{k_2^2}{(k_1 + k_2)^2} + \left(1 - \frac{k_2^2}{(k_1 + k_2)^2}\right)} = 1$

At t --> infinity: $\frac{M_n}{M} \rightarrow \frac{\frac{k_1}{k_1 + k_2}}{\left(1 - \frac{k_2^2}{(k_1 + k_2)^2}\right)} = \frac{k_1^2 + k_1 k_2}{k_1^2 + 2 k_1 k_2} = \frac{k_1 + k_2}{k_1 + 2 k_2}$

Depolymerization conversion:

$$\%depol = \frac{[AA]_0 - [AA] - [AC] - [CC] - \frac{[A] + [C]}{2}}{[AA]_0}$$

$$\%depol = 1 - \mathrm{e}^{-(\mathrm{k}_1+\mathrm{k}_2)t} - k_1 t e^{-(k_1+k_2)t} - \frac{k_1^2}{(k_1 + k_2)^2}\left(1 - (1 + (k_1 + k_2)t) e^{-(k_1+k_2)t}\right) - \frac{k_2}{2} t e^{-(k_1+k_2)t} - \frac{k_1 k_2}{(k_1 + k_2)^2} 1 - (1 + (k_1 + k_2)t) e^{-(k_1+k_2)t}\big)$$

$$\%depol = 1 - \frac{k_1^2}{(k_1 + k_2)^2} - \frac{k_1 k_2}{(k_1 + k_2)^2} - \left(1 - \frac{k_1^2}{(k_1 + k_2)^2} - \frac{k_1 k_2}{(k_1 + k_2)^2}\right) \mathrm{e}^{-(k_1+k_2)t} - \left(k_1 + \frac{k_2}{2}\right) t e^{-(k_1+k_2)t} + \frac{k_1^2 + k_1 k_2}{(k_1 + k_2)^2}(k_1 + k_2) t e^{-(k_1+k_2)t}$$

$$\%depol = \frac{k_2^2 + k_1 k_2}{(k_1 + k_2)^2}(1 - \mathrm{e}^{-(k_1+k_2)t}) + \left(\frac{k_1^2 + k_1 k_2}{k_1 + k_2} - \left(k_1 + \frac{k_2}{2}\right)\right) t e^{-(k_1+k_2)t}$$

$$\%depol = \frac{k_2^2 + k_1 k_2}{(k_1 + k_2)^2}(1 - \mathrm{e}^{-(k_1+k_2)t}) + \left(\frac{k_1^2 + k_1 k_2 - k_1^2 - k_1 k_2 - \frac{k_1 k_2}{2} - \frac{k_2^2}{2}}{k_1 + k_2}\right) t e^{-(k_1+k_2)t}$$

$$\%depol = \frac{k_2}{k_1 + k_2}(1 - \mathrm{e}^{-(k_1+k_2)t}) - \frac{k_2}{2} t e^{-(k_1+k_2)t}$$

%UV variation during the depolymerization

$$\%UV = \frac{[AA] + \frac{[AC]}{2} + \frac{[A]}{2}}{[AA]_0}$$

$$\%UV = \mathrm{e}^{-(k_1+k_2)t} + \frac{k_1}{2} te^{-(k_1+k_2)t} + \frac{k_2}{2} te^{-(k_1+k_2)t}$$

$$\%UV = \left(1 + \frac{k_1 + k_2}{2} t\right) e^{-(k_1+k_2)t}$$

3.2. Mathematical model for R-terminal bifunctional polymers

Species:

A: initial polymer, $M_n=M$

B: living polymer after either depolymerization or termination of one arm, $M_n = M/2$

C: dead polymer, $M_n = M/2$

Reaction scheme:

A --> B + C (termination, rate coefficient k_1)

A --> B+D (depolymerization, rate coefficient k_2)

B --> C (termination, rate coefficient k_1)

B --> D (depolymerization, rate coefficient k_2)

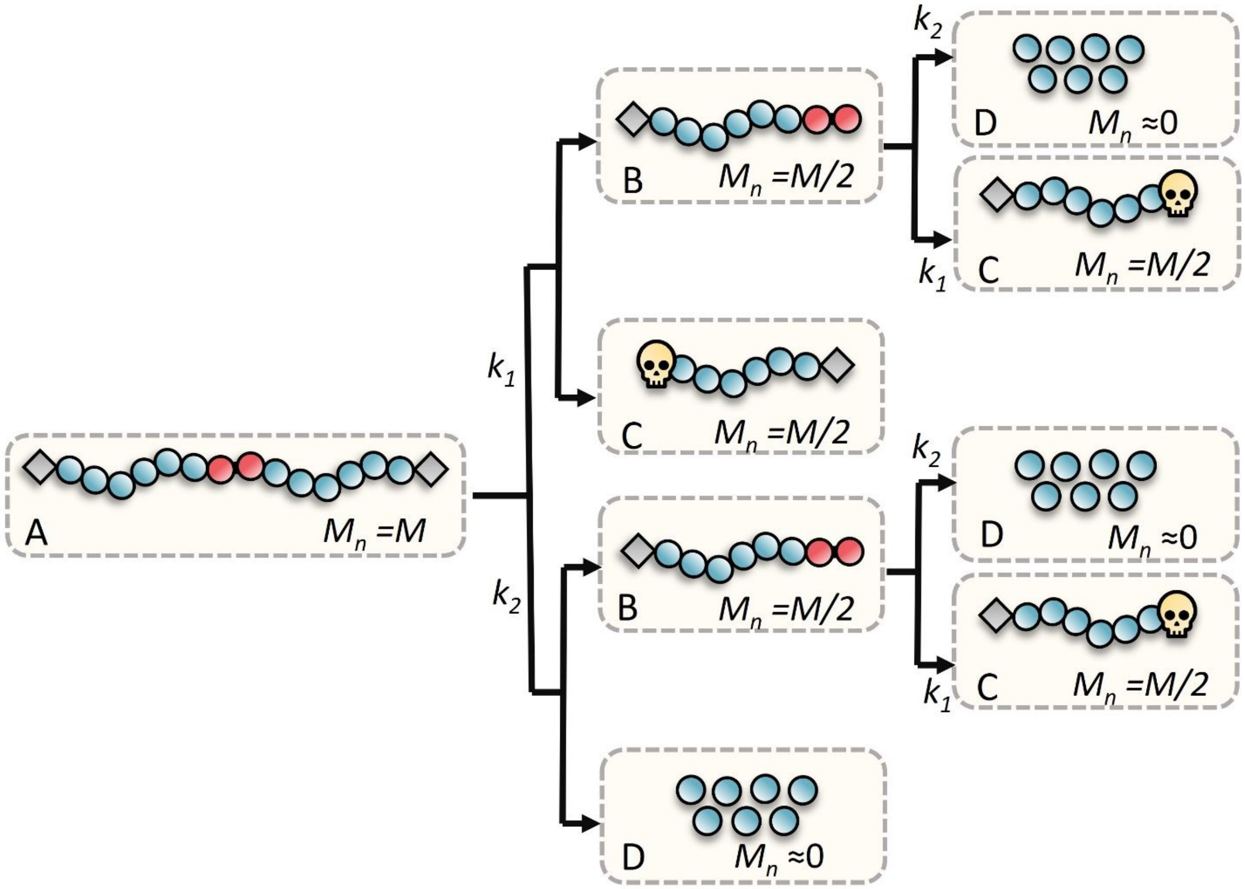

Scheme S7: Schematic diagram describing the events during the depolymerization of the R-bifunctional polymers: A: Initial polymer (M_n = M), B : living polymer after depolymerization of one arm, (M_n = M/2), C : dead polymer after depolymerization of one arm, (M_n = M/2), D : Depolymerization of both branches $M_n \sim 0$, k_1=rate of termination, k_2=rate of depolymerization

Kinetics:

$$\frac{-d[A]}{dt} = k_1[A] + k_2[A]$$

$$\frac{d[B]}{dt} = k_1[A] + k_2[A] - k_1[B] - k_2[B]$$

$$\frac{d[C]}{dt} = k_1[A] + k_1[B]$$

Solving the system of differential equations gives:

$$[A] = [A]_0 \mathrm{e}^{-(k_1+k_2)t}$$

$$\frac{d[B]}{dt} + (k_1 + k_2)[B] = (k_1 + k_2)[A]$$

$$\frac{d}{dt}(\mathrm{e}^{(k_1+k_2)t}[B]) = \mathrm{e}^{(k_1+k_2)t}(k_1 + k_2)[A]_0 \mathrm{e}^{-(k_1+k_2)t} = (k_1 + k_2)[A]_0$$

$$[B] = (k_1 + k_2)[A_0]t\mathrm{e}^{-(k_1+k_2)t} + const$$

when t = 0, [B] = 0 --> const. = 0 :

$$[B] = (k_1 + k_2)[A_0]t\mathrm{e}^{-(k_1+k_2)t}$$

$$\frac{d[C]}{dt} = k_1[A]_0\mathrm{e}^{-(k_1+k_2)t}(1 + (k_1 + k_2)t)$$

$$[C] = \frac{k_1[A]_0}{k_1 + k_2}\left(- \mathrm{e}^{-(k_1+k_2)t} - (k_1 + k_2)t\, e^{-(k_1+k_2)t} - e^{-(k_1+k_2)t} + c\right)$$

$$[C] = \frac{k_1[A]_0}{k_1 + k_2}\left(-2\mathrm{e}^{-(k_1+k_2)t} - (k_1 + k_2)t\, e^{-(k_1+k_2)t} + c\right)$$

when t = 0, [C] = 0

$$c = \frac{2\, k_1[A]_0}{k_1 + k_2}$$

$$[C] = \frac{k_1[A]_0}{k_1 + k_2}\left(2 - 2\mathrm{e}^{-(k_1+k_2)t} - (k_1 + k_2)t\, e^{-(k_1+k_2)t}\right)$$

$$[C] = \frac{2\, k_1[A]_0}{k_1 + k_2}(1 - \mathrm{e}^{-(k_1+k_2)t}) - k_1[A]_0 t\, e^{-(k_1+k_2)t}$$

Average MW:

[B] + [C] :

$$[B] + [C] = (k_1 + k_2)[A_0]t\mathrm{e}^{-(k_1+k_2)t} + \frac{2\, k_1[A]_0}{k_1 + k_2}(1 - \mathrm{e}^{-(k_1+k_2)t}) - k_1[A]_0 t\, e^{-(k_1+k_2)t}$$

$$[B] + [C] = \frac{2\, k_1[A]_0}{k_1 + k_2}(1 - \mathrm{e}^{-(k_1+k_2)t}) + k_2[A]_0 t\, e^{-(k_1+k_2)t}$$

[A] + [B] + [C] :

$$[A] + [B] + [C] = [A]_0\mathrm{e}^{-(k_1+k_2)t} + \frac{2\, k_1[A]_0}{k_1 + k_2}(1 - \mathrm{e}^{-(k_1+k_2)t}) + k_2[A]_0 t\, e^{-(k_1+k_2)t}$$

$$[A] + [B] + [C] = \frac{2\, k_1[A]_0}{k_1 + k_2} + \frac{(k_2 - k_1)[A]_0}{k_1 + k_2}\mathrm{e}^{-(k_1+k_2)t} + k_2[A]_0 t\, e^{-(k_1+k_2)t}$$

M_n :

$$M_n = M\left(1 - \frac{\frac{[B] + [C]}{2}}{[A] + [B] + [C]}\right)$$

$$M_n = M\left(1 - \frac{k_1(\mathrm{e}^{(k_1+k_2)t} - 1) + \frac{k_2(k_1 + k_2)}{2}t}{2k_1\mathrm{e}^{(k_1+k_2)t} + (k_2 - k_1) + k_2(k_1 + k_2)t}\right)$$

$$M_n = M\left(\frac{2k_1\mathrm{e}^{(k_1+k_2)t} + (k_2 - k_1) + k_2(k_1 + k_2)t - k_1(\mathrm{e}^{(k_1+k_2)t} - 1) - \frac{k_2(k_1 + k_2)}{2}t}{2k_1\mathrm{e}^{(k_1+k_2)t} + (k_2 - k_1) + k_2(k_1 + k_2)t}\right)$$

$$M_n = M\left(\frac{2k_1\mathrm{e}^{(k_1+k_2)t} + (k_2 - k_1) + k_2(k_1 + k_2)t - k_1\mathrm{e}^{(k_1+k_2)t} + k_1 - \frac{k_2(k_1 + k_2)}{2}t}{2k_1\mathrm{e}^{(k_1+k_2)t} + (k_2 - k_1) + k_2(k_1 + k_2)t}\right)$$

$$M_n = M\left(\frac{k_1\mathrm{e}^{(k_1+k_2)t} + k_2 + \frac{k_2(k_1 + k_2)}{2}t}{2k_1\mathrm{e}^{(k_1+k_2)t} + (k_2 - k_1) + k_2(k_1 + k_2)t}\right)$$

$$M_n = \frac{M}{2}\left(\frac{2k_1\mathrm{e}^{(k_1+k_2)t} + 2k_2 + k_2(k_1 + k_2)t}{2k_1\mathrm{e}^{(k_1+k_2)t} + (k_2 - k_1) + k_2(k_1 + k_2)t}\right)$$

$$M_n = \frac{M}{2}\left(\frac{2k_1\mathrm{e}^{(k_1+k_2)t} + (k_2 - k_1) + k_2(k_1 + k_2)t + (k_1 + k_2)}{2k_1\mathrm{e}^{(k_1+k_2)t} + (k_2 - k_1) + k_2(k_1 + k_2)t}\right)$$

$$M_n = \frac{M}{2}\left(1 + \frac{(k_1 + k_2)}{2k_1 e^{(k_1+k_2)t} + (k_2 - k_1) + k_2(k_1 + k_2)t}\right)$$
$$M_n = \frac{M}{2}\left(1 + \frac{(k_1 + k_2)\, e^{-(k_1+k_2)t}}{2k_1 + (k_2 - k_1)e^{-(k_1+k_2)t} + k_2(k_1 + k_2)te^{-(k_1+k_2)t}}\right)$$

$$M_n \rightarrow \frac{M}{2}\left(1 + \frac{(k_1 + k_2)\, e^{-(k_1+k_2)t}}{2k_1}\right) \rightarrow \frac{M}{2}$$

Depolymerization conversion:

$$\%depol = \frac{[A]_0 - [A] - \frac{[B] + [C]}{2}}{[A]_0}$$
$$\%depol = 1 - e^{-(k_1+k_2)t} - \frac{k_1}{k_1 + k_2}(1 - e^{-(k_1+k_2)t}) - \frac{k_2}{2}\, t\, e^{-(k_1+k_2)t}$$
$$\%depol = \frac{k_2}{k_1 + k_2}(1 - e^{-(k_1+k_2)t}) - \frac{k_2}{2}\, t\, e^{-(k_1+k_2)t}$$
$$\%depol \rightarrow \frac{k_2}{k_1 + k_2}$$

%UV variation during the depolymerization:

$$\%UV = \frac{[A] + \frac{[B]}{2}}{[A]_0}$$

$$\%UV = \left(1 + \frac{(k_1 + k_2)}{2}\, t\right) e^{-(k_1+k_2)t}$$

4. Supplementary Figures:

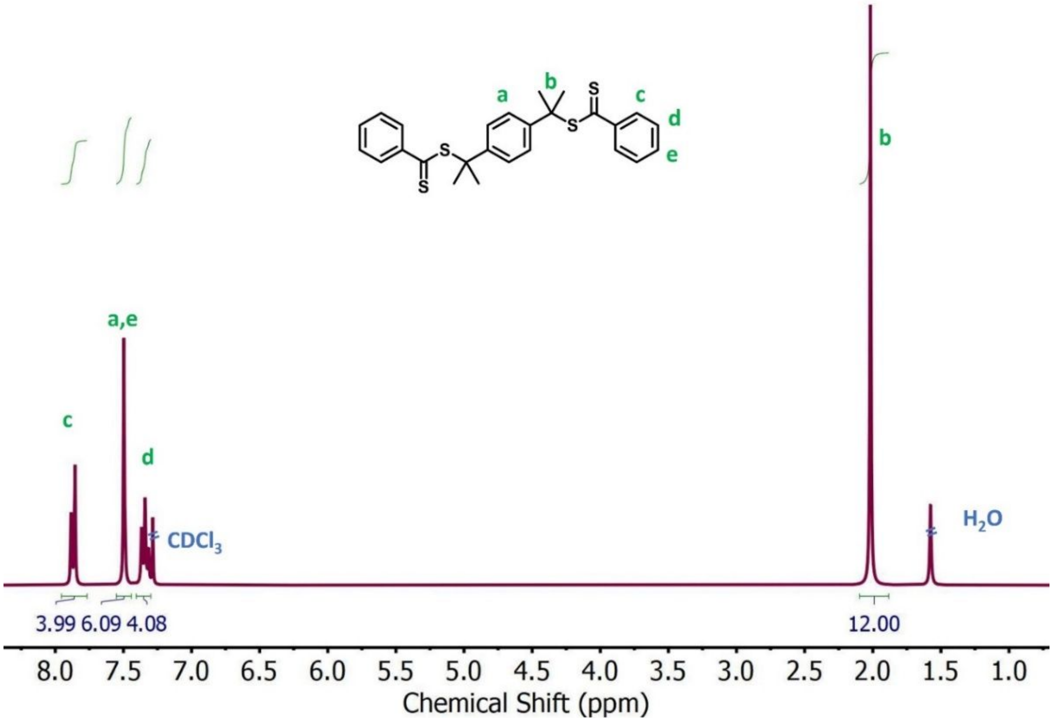

Figure S1: ^{1}H-NMR spectra of Z-terminal bifunctional CTA

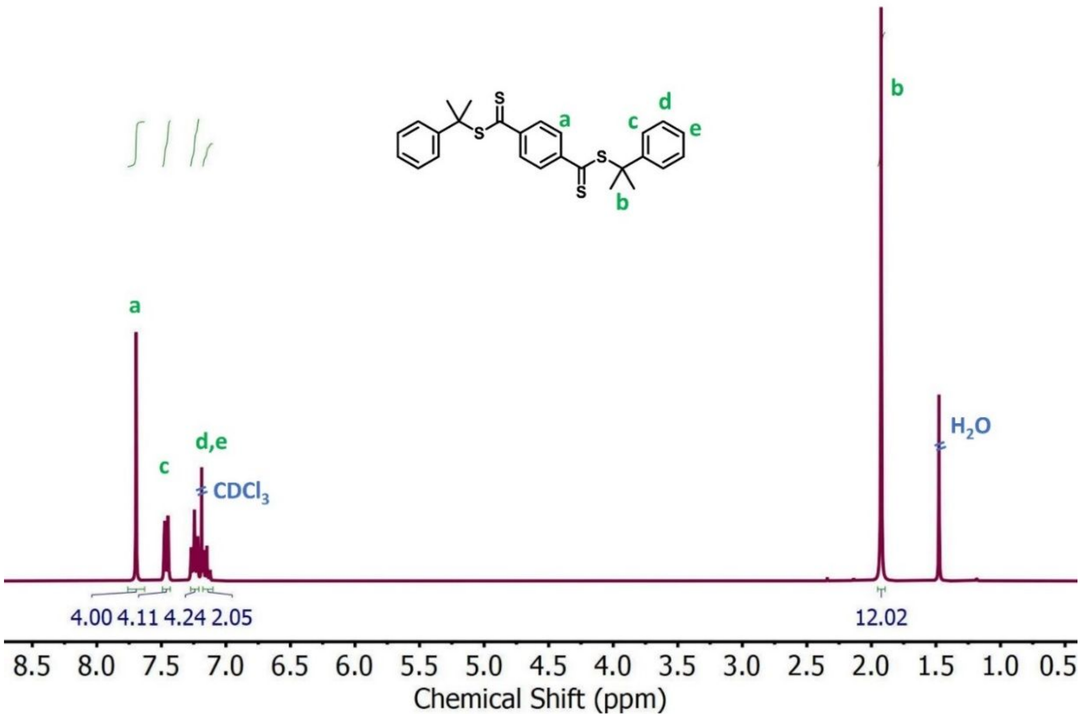

Figure S2: ^{1}H-NMR spectra of R-terminal bifunctional CTA

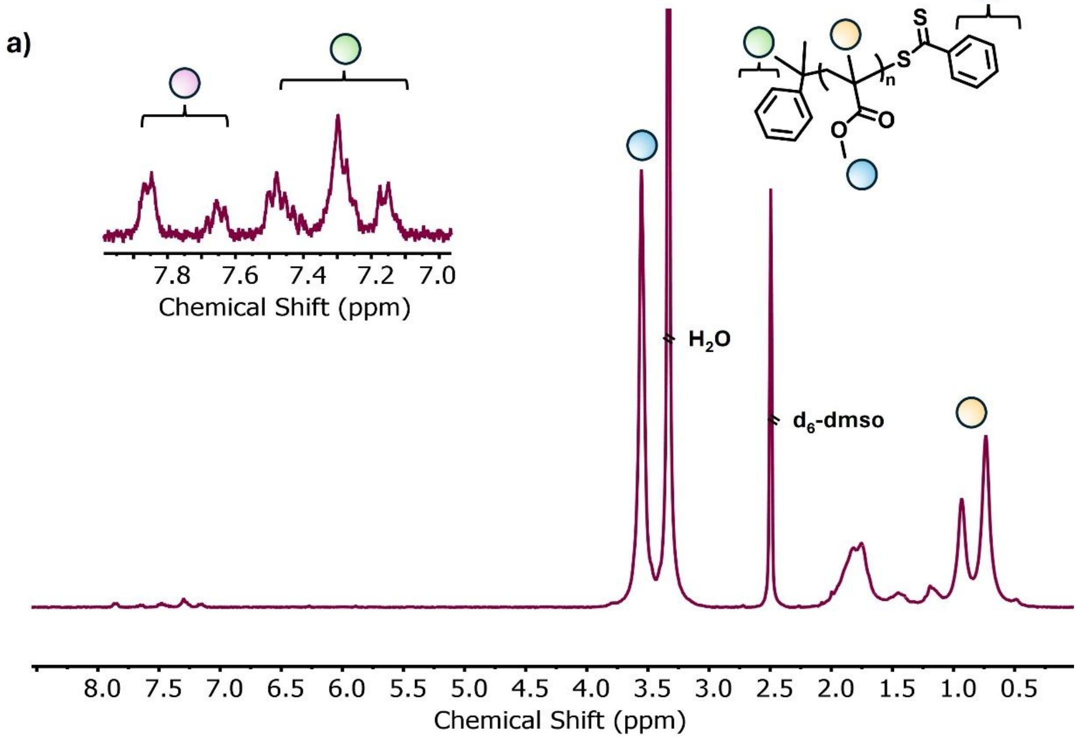

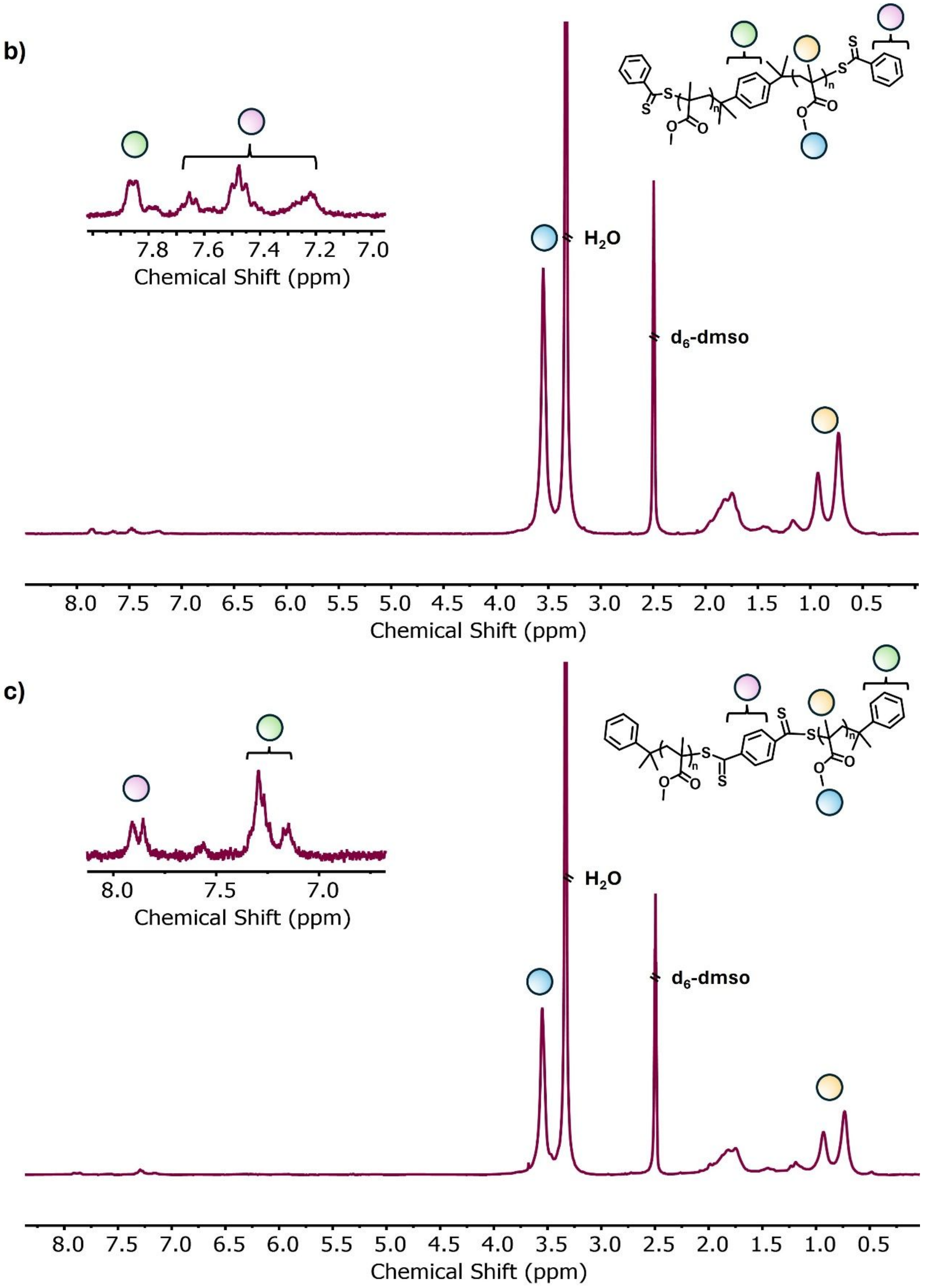

Figure S3: ^{1}H-NMR spectra of polymers synthesized with a). cumyl dithiobenzoate CTA, b). Z-terminal bifunctional CTA, c). R-terminal bifunctional CTA.

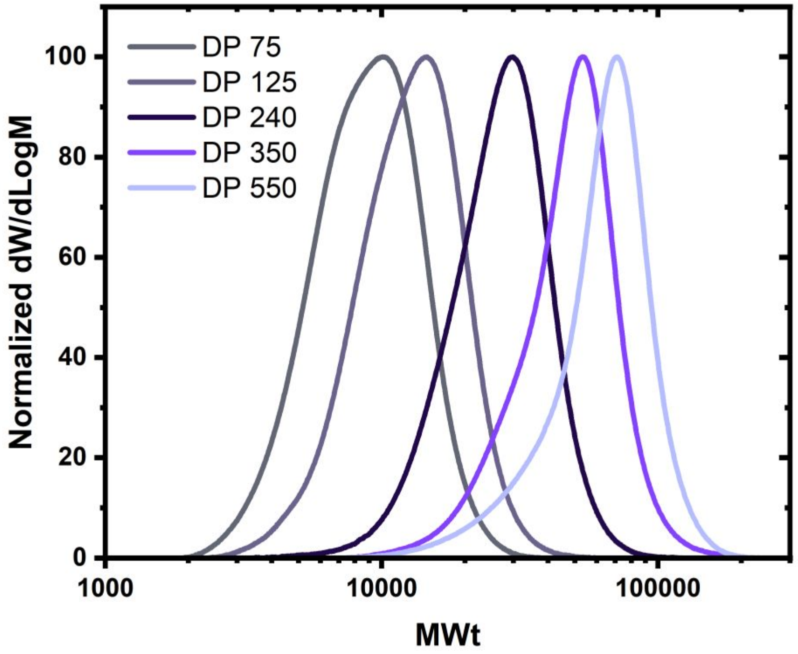

Figure S4: SEC traces of polymers synthesized with Z-terminal bifunctional CTA

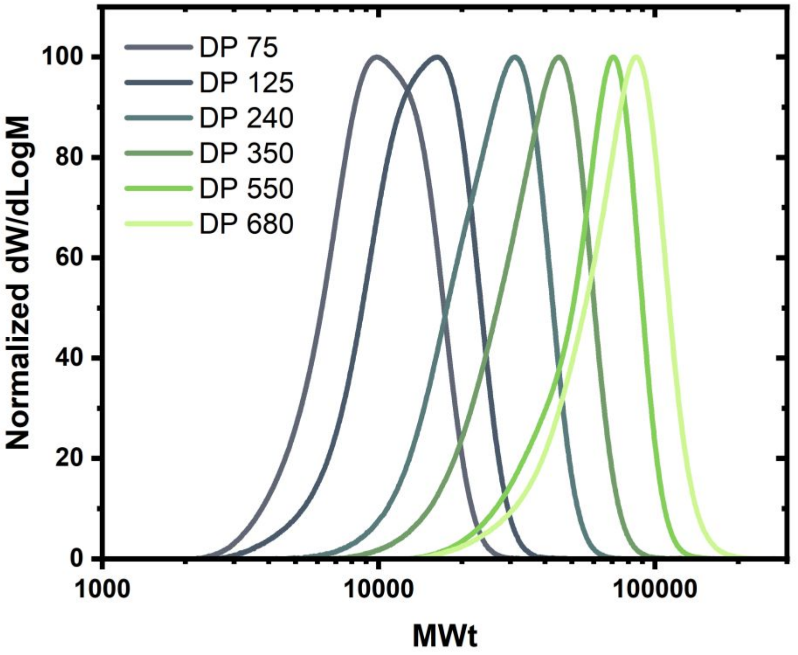

Figure S5: SEC traces of polymers synthesized with R-terminal bifunctional CTA

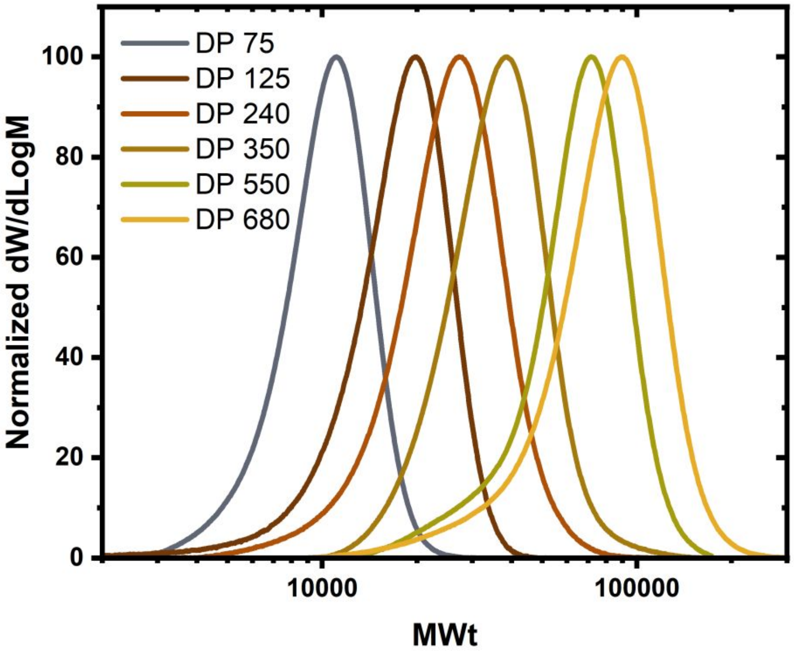

Figure S6: SEC traces of polymers synthesized with cumyl dithiobenzoate CTA

Table S 1: M_n and $Đ$ values of monofunctional, Z-terminal bifunctional and R-terminal bifunctional polymers

DP	Monofunctional polymers		Z-terminal bifunctional polymers		R-terminal bifunctional polymers	
	M_n	$Đ$	M_n	$Đ$	M_n	$Đ$
**75**	8000	1.22	7600	1.19	7800	1.18
**125**	12300	1.14	12600	1.16	12300	1.17
**240**	24800	1.11	24100	1.19	23700	1.16
**350**	35200	1.14	34800	1.20	34600	1.16
**550**	55700	1.17	54800	1.12	55100	1.14
**680**	68200	1.23	-	-	67800	1.14

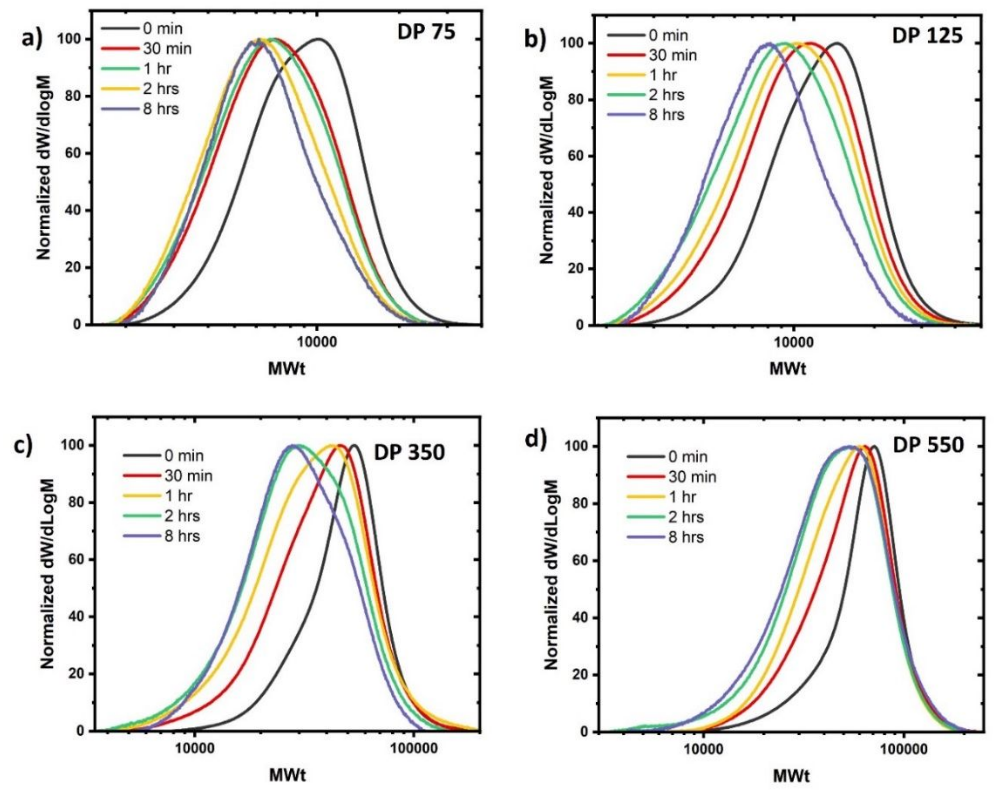

Figure S7: SEC traces obtained during the depolymerization of Z-terminal bifunctional polymers: a). DP 75, b). DP 125, c). DP 350 and d). DP 550.

Table S 2: Depolymerization conversions, M_n values (experimental) and M_n shifts for the depolymerization of the DP 240 Z-terminal bifunctional polymer and monofunctional polymer.

Time (min)	Depo% (Z-terminal bifunc)	$M_{n(exp)}$ (Z-terminal bifunc)	M_n shift (Z-terminal bifunc) %	Depo% (mono)	$M_{n(exp)}$ (mono)	M_n shift (mono) %
0	0	24100	0	0	24800	0
15	16	20000	17	25	22700	8
30	24	18400	24	58	22100	11
60	45	16900	30	61	22000	11
120	63	14600	40	67	21600	13
240	74	13400	44	68	20300	18
480	80	13000	46	68	19800	20

*Depo% = (Area of RI signal at t_0- Area of RI signal at t)/ Area of RI signal at t_0

*M_n shift=($M_{n(0)}$- $M_{n(t)}$)/ $M_{n(0)}$, ($M_{n(0)}$=M_n at time 0, $M_{n(t)}$=M_n at time t)

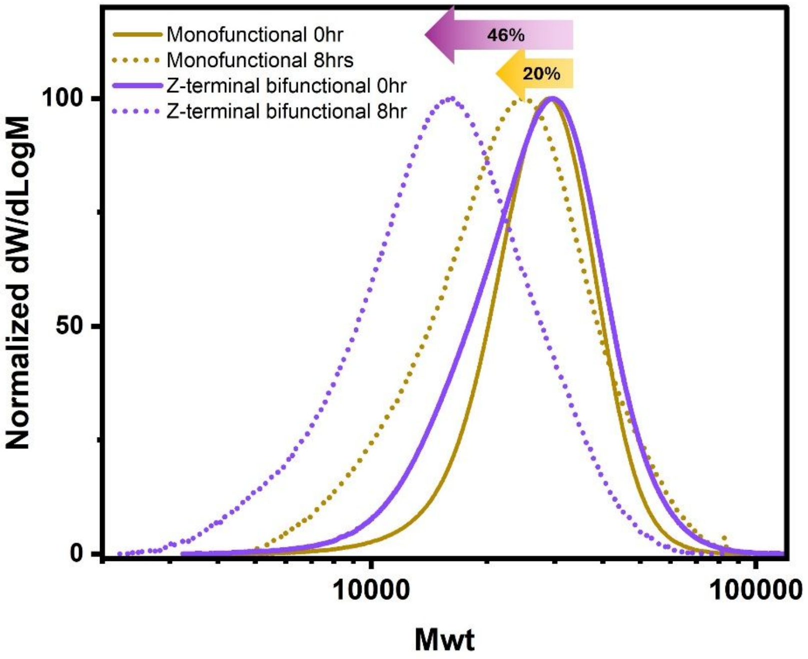

Figure S8: SEC traces demonstrating the M_n Shift which occurred during the depolymerization of DP 240 monofunctional (yellow) and Z-terminal bifunctional (purple) polymers.

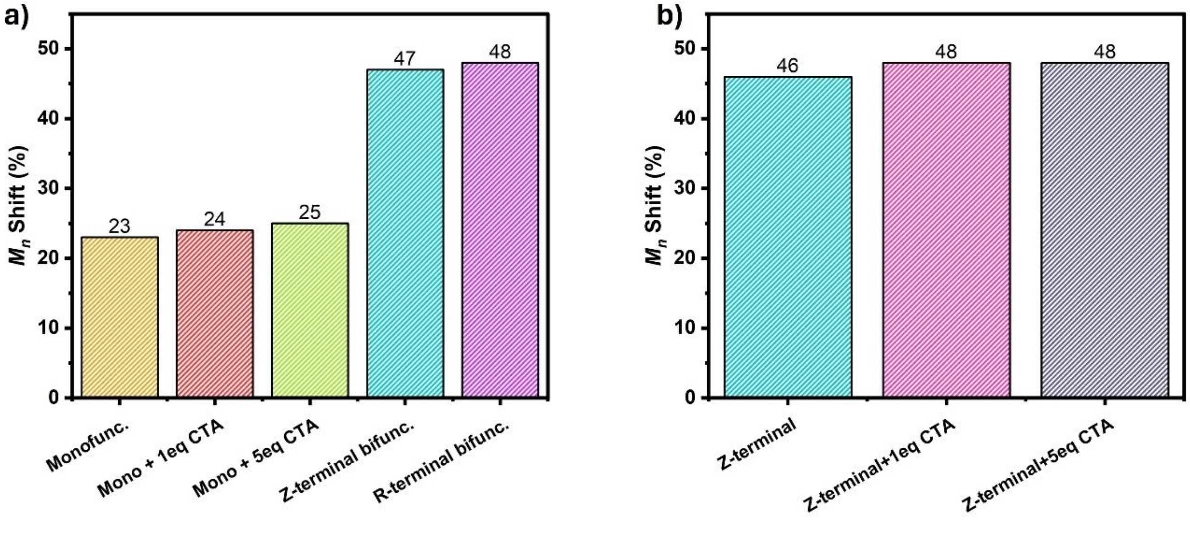

Figure S9: a). Bar graph representing the M_n shifts of (reaction conditions: 5 mM repeating unit concentration of polymer in dioxane at 120 °C) DP 240 monofunctional polymer (yellow), DP 240 monofunctional polymer with 1 eq. of CTA (red), DP 240 monofunctional polymer with 5 eq. of CTA (green), DP 240 Z-terminal bifunctional polymer (blue), DP 240 R-terminal bifunctional polymer (purple), b) Bar graph representing the M_n shifts of (reaction conditions: 5 mM repeating unit concentration of polymer in dioxane at 120 °C) DP 240 Z-terminal bifunctional polymer (blue), DP 240 Z-terminal bifunctional polymer with 1 eq. of CTA (pink), DP 240 Z-terminal bifunctional polymer with 5 eq. of CTA (grey).

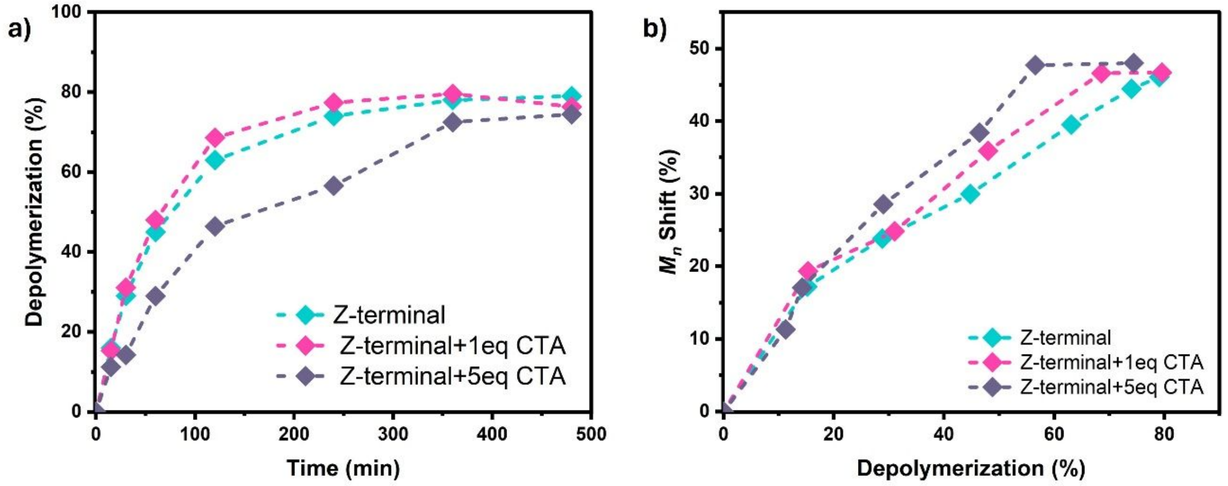

Figure S10: a). Depolymerization kinetics of the Z-terminal bifunctional polymer (reaction conditions: 5 mM repeating unit concentration of polymer in dioxane at 120 °C) with addition of 0, 1 and 5 eq. of CTA, b). M_n shifts comparison of the depolymerization reactions with different amount of CTA addition.

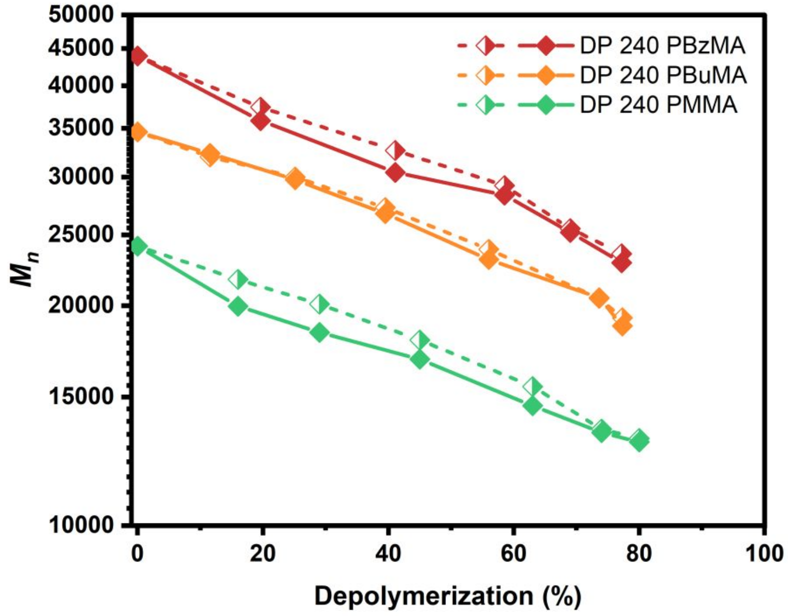

Figure S11: Comparison between the modeled (half-filled diamonds) and experimentally obtained (filled diamonds) M_n values during the depolymerization of poly(benzyl methacrylate, poly(butyl methacrylate) and poly(methyl methacrylate) Z-terminal bifunctional polymer. (solid lines represent the variation of $M_{n(exp)}$, where the dashed lined represent the variation of $M_{n(model)}$)

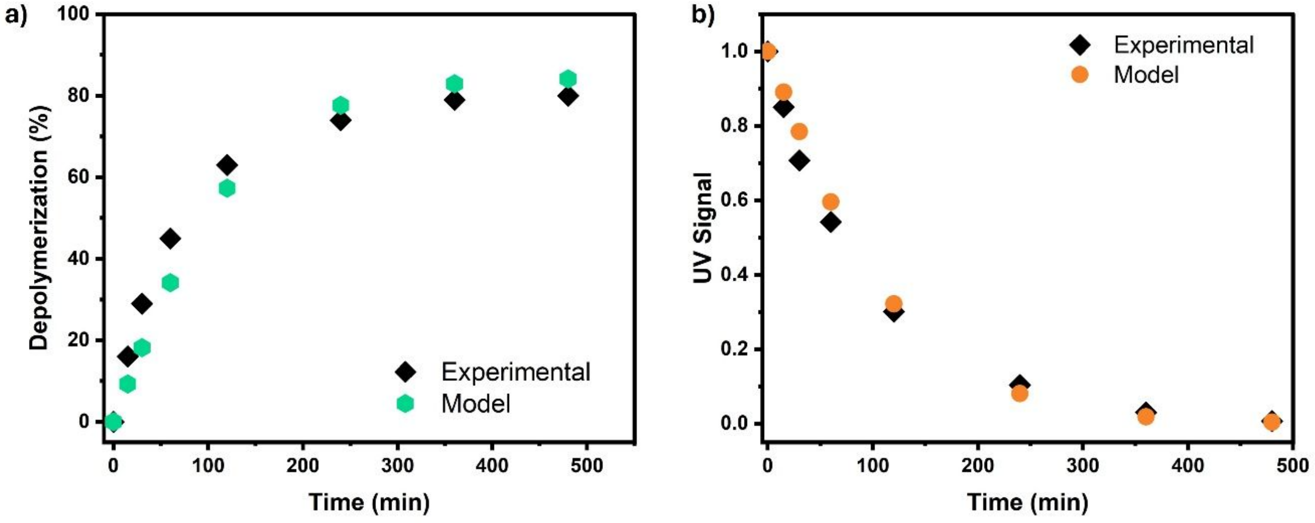

Figure S12: a). experimental and model kinetics for the depolymerization DP 240 Z-terminal polymers, b). experimental and model UV signal corresponding for the depolymerization DP 240 Z-terminal polymers.

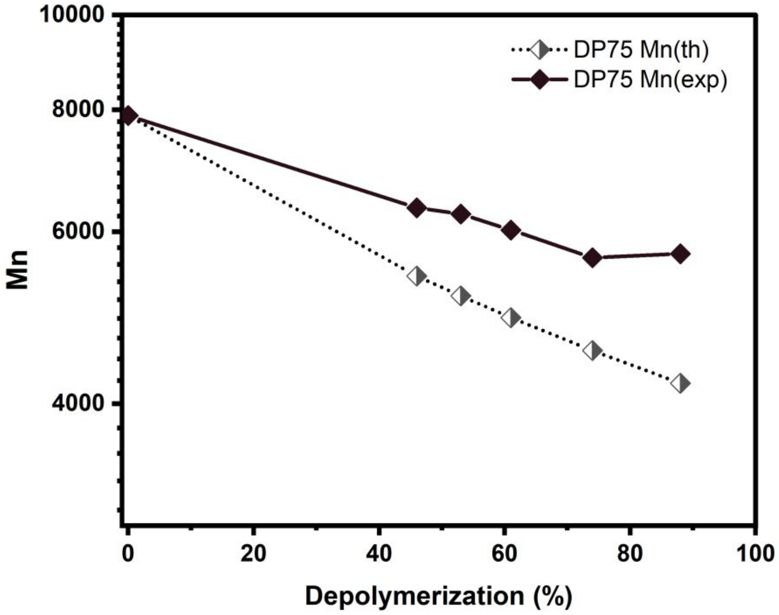

Figure S13 : Comparison between the modeled and experimentally obtained M_n values during the depolymerization of DP 75 Z-terminal bifunctional polymer.

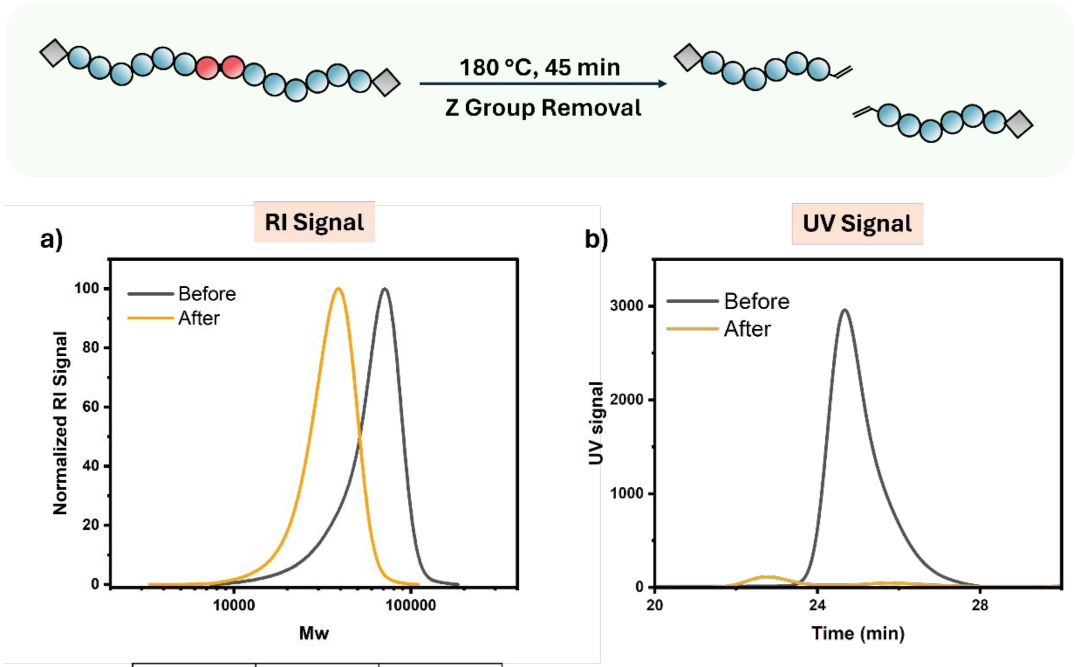

	M_n	Đ
**Before**	53200	1.18
**After**	30600	1.13

Figure S14: End group removal of DP 550 R-terminal bifunctional polymer (conditions: the solid polymer heated at 180 °C for 45 min) a). Normalized RI signal before and after end group removal, b). UV signal before and after the end group removal. (Typically, during thermolysis, there should be no noticeable shift in the SEC traces before and after the process, as only the RAFT Z groups are removed. However, the significant shift observed in the SEC results can be attributed to a unique aspect of the R-terminal bifunctional polymers. In these polymers, the Z groups are situated in the middle of the chain. Upon the removal of these Z groups, the chain undergoes cleavage into two halves, each with half the original length. This cleavage results in a substantial alteration in the apparent molecular weight of the polymer, as detected by SEC analysis.)

Table S3: Comparison between the depolymerization conversions, M_n values (experimental) and M_n shifts of DP 240 R-terminal bifunctional polymer and DP 240 Z-terminal bifunctional polymers.

Time (min)	Depo% (R-terminal bifunc)	$M_{n(exp)}$ (R-terminal bifunc)	M_n shift (R-terminal bifunc) (exp) %	Depo% (Z-terminal bifunc)	$M_{n(exp)}$ (Z-terminal bifunc)	M_n shift (Z-terminal bifunc) (exp) %
0	0	23700	0	0	24100	0
15	22	17600	26	16	20000	17
30	38	15400	35	24	18400	24
60	63	12400	48	45	16900	30
120	78	12500	48	63	14600	40
240	81	12400	48	74	13400	44
480	84	12100	49	80	13000	46

*Depo% = (Area of RI signal at t_0- Area of RI signal at t)/ Area of RI signal at t_0

*M_n shift=($M_{n(0)}$- $M_{n(t)}$)/ $M_{n(0)}$, ($M_{n(0)}$=M_n at time 0, $M_{n(t)}$=M_n at time t)

DP	Z-terminal polymers		R-terminal polymers	
	**Estimated k_1 (min^{-1})**	**Estimated k_2 (min^{-1})**	**Estimated k_1 (min^{-1})**	**Estimated k_2 (min^{-1})**
**75**	0.0180	0.0420	0.0023	0.0143
**125**	0.0033	0.0180	0.0038	0.0244
**240**	0.0023	0.0124	0.0071	0.0317
**350**	0.0080	0.0165	0.0141	0.0431
**550**	0.0213	0.0246	0.0257	0.0500
**680**	-	-	0.0375	0.0368

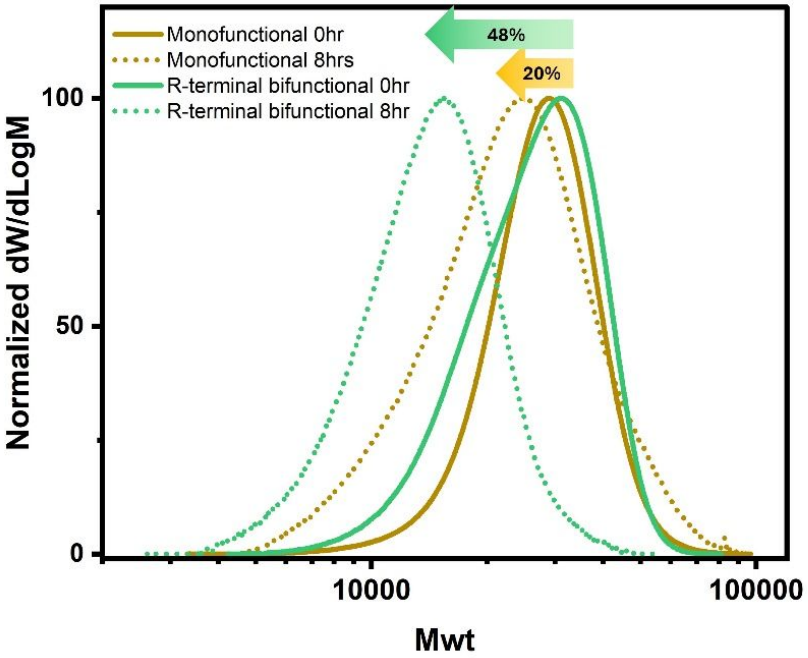

Figure S15: M_n shift during the depolymerization for DP 240 monofunctional (yellow) and R-terminal bifunctional (green) polymers.

Table S4: Estimated k_1 and k_2 values for Z-terminal polymers and R-terminal polymers using the model.

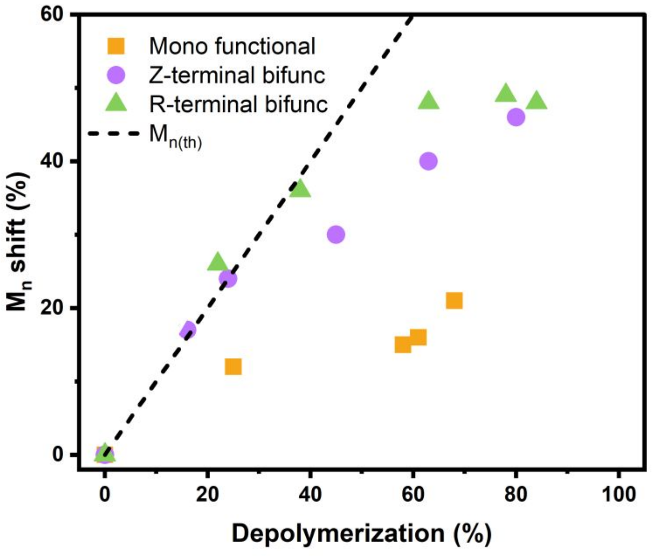

Figure S16: M_n shifts vs depolymerization conversions for various DP 240 polymers (reaction conditions: 5 mM repeating unit concentrations in dioxane at 120 °C) yellow: monofunctional, purple: Z-terminal bifunctional polymer, green: R-terminal bifunctional polymer.

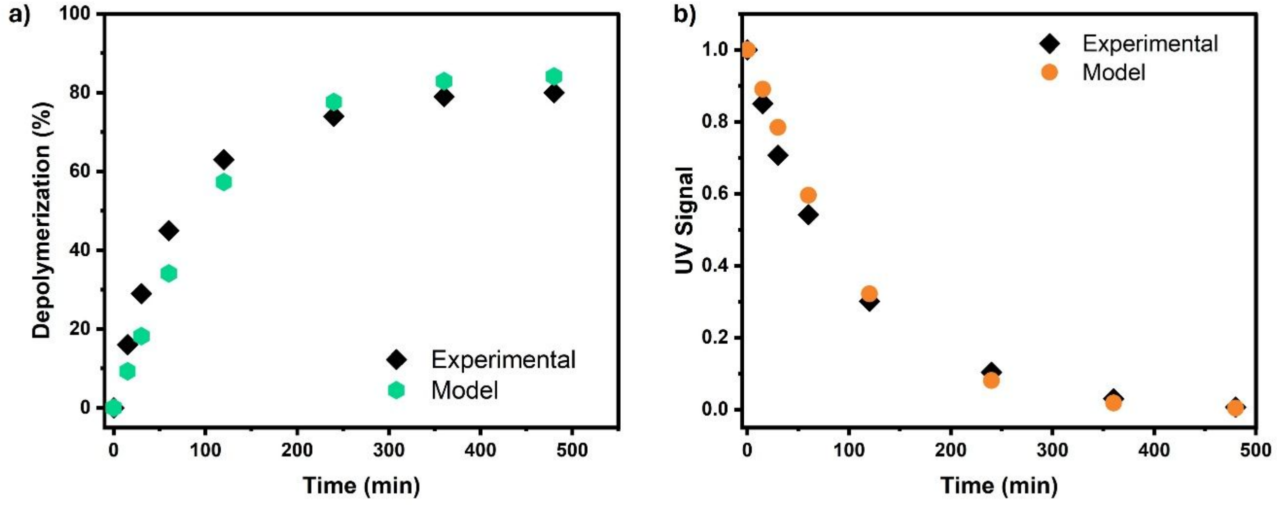

Figure S17:a). experimental and model kinetics for the depolymerization DP 240 Z-terminal polymers, b). experimental and model UV signal changes for the depolymerization DP 240 Z-terminal polymers.

References:

(1) L. Patton, D.; Mullings, M.; Fulghum, T.; C. Advincula, R. A Facile Synthesis Route to Thiol-Functionalized α,ω-Telechelic Polymers via Reversible Addition Fragmentation Chain Transfer Polymerization. *Macromolecules* **2005**, *38* (20), 8597–8602. https://doi.org/10.1021/ma051035s.

(2) Guice, K. B.; Marrou, S. R.; Gondi, S. R.; Sumerlin, B. S.; Loo, Y.-L. PH Response of Model Diblock and Triblock Copolymer Networks Containing Polystyrene and Poly(2-Hydroxyethyl Methacrylate- *Co* -2-(Dimethylamino)Ethyl Methacrylate). *Macromolecules* **2008**, *41* (12), 4390–4397. https://doi.org/10.1021/ma8003746.
